# Supplementary material for: The radiation continuum and the evolution of frog diversity
Source: Nat Commun. 2023 Nov 4;14:7100. doi: 10.1038/s41467-023-42745-x (PMC10625520; doi:10.1038/s41467-023-42745-x)
Supplement: Supplementary file 1 — Supplementary Information [file 41467_2023_42745_MOESM1_ESM.pdf]

Supplementary Information for

The radiation continuum and the evolution of frog diversity

Gen Morinaga[1,2], John J. Wiens[3], and Daniel S. Moen[1,4,*]

[1]Department of Integrative Biology, Oklahoma State University, Stillwater, OK 74078, USA. [2]Department of Comparative Biology, University of Calgary, Calgary, Alberta T2N 4N1, Canada. [3]Department of Ecology and Evolutionary Biology, University of Arizona, Tucson, AZ 85721, USA. [4]Department of Evolution, Ecology, and Organismal Biology, University of California, Riverside, Riverside, CA 92521, USA.

*Corresponding author: dmoen@ucr.edu

This file includes:

Supplementary Tables 1–5

Supplementary Figures 1–7

Supplementary Table 1 | Eigenvector coefficients for the first five principal component axes from the phylogenetic principal components analysis (pPCA).

Original variables	pPC-1	pPC-2	pPC-3	pPC-4	pPC-5
Head length	0.114	-0.089	-0.037	-0.377	0.797
Head width	-0.026	0.039	0.206	-0.391	0.606
Front limb length	0.255	-0.175	-0.034	-0.298	0.474
Hindlimb length	0.325	-0.012	0.110	-0.648	0.129
Foot webbing area	0.872	0.485	-0.053	0.042	0.001
Foot adhesive pad area	0.592	-0.743	-0.130	-0.062	-0.079
Hand adhesive pad area	0.568	-0.765	-0.183	0.046	0.037
Inner metatarsal tubercle area	0.347	-0.279	0.847	0.278	0.078
Thigh volume	0.314	0.140	0.324	-0.692	-0.052
Crus volume	0.233	-0.094	0.463	-0.659	-0.400
Eigenvalues	0.006	0.004	0.002	0.001	0.001
Proportion of variance explained	0.412	0.282	0.115	0.072	0.043
Total proportion of variance explained	0.412	0.694	0.809	0.881	0.924

pPCA used a covariance matrix and assumed a Brownian motion model of evolution. We size-corrected and ln-transformed all variables prior to analysis. We also took the square-roots and cube-roots of the area and volume measurements, respectively, prior to size correction. Source Data can be found within Supplementary Code 1.

Supplementary Table 2 | Phylogenetic generalized least squares correlations (assuming Brownian motion) between various variables and the diversification rates estimated using birth-death estimators.

	r	*P*
ln (Species #)	0.738	2.10×10^{-9}
Morphological rate	0.237	0.12613
Crown (ε = 0)	0.741	1.74×10^{-9}
Crown (ε = 0.5)	0.751	6.70×10^{-10}
Crown (ε = 0.9)	0.767	1.46×10^{-10}
Stem (ε = 0)	0.768	1.29×10^{-10}
Stem (ε = 0.5)	0.771	9.47×10^{-11}
Stem (ε = 0.9)	0.781	3.58×10^{-11}

ε is the extinction fraction assumed for diversification rates estimated using the method-of-moments estimator. All *P*-values reflect two-sided hypothesis tests with no adjustment for multiple comparisons. Source Data can be found within Supplementary Code 1.

Supplementary Table 3 | Results from tests of phylogenetic clustering of radiation-space types across 43 anuran families.

Quadrant	D	P_0	P_1
Adaptive radiation	0.731	0.235	0.299
Adaptive non-radiation	0.500	0.356	0.268
Non-adaptive, non-radiation	0.284	0.459	0.169
Non-adaptive radiation	-0.351	0.648	0.088

D is the *D*-statistic of phylogenetic signal for binary traits[45]. We assessed each quadrant type individually, where states of binary trait were that quadrant type versus all others (e.g., states of a binary trait for adaptive radiation were "adaptive radiation" vs. "not adaptive radiation"). P_0 is the probability of obtaining the estimated *D* or one farther from 0 if the true *D* were 0 (i.e., phylogenetic clustering). P_1 is the analogous probability under a true *D* of 1 (i.e., random distribution across the tips). In both cases, the *P*-values were compared to α = 0.05, showing that none of the quadrant types were significantly different than 0 or 1. Source Data can be found within Supplementary Code 1.

Supplementary Table 4 | Detailed descriptions of morphometric measurements.

Measurement	Description
Snout–vent length	Length measured from tip of the snout to the cloaca while viewing the animal from the dorsal side
Head length	Length measured from tip of the snout to the posterior-most part of the mouth while viewing the animal dorso-laterally.
Head width	Length measured transversely, from either lateral side of the posterior-most position of the mouth to the other while viewing the animal from the dorsal side.
Upper front limb length	Length measured from the axilla, where the arm exits the body, to the tip of the elbow, while viewing the animal from the ventral side.
Forearm length	Length measured from the tip of the elbow to the base of the thumb on the manus, while viewing the animal from the ventral side
Hand length	Length measured from the proximal-most base of the thumb to the distal tip of the longest front limb digit, while viewing the animal from the ventral side
Thigh length	Length measured from the cloaca to the tip of the knee while viewing the animal from the ventral side
Crus length	Length measured from the tip of the knee to the proximal-most position of the tarsus, at the ankle. We identified the ankle as the point where more distal segments of the hind limb bend. We measured this while viewing the animal from the ventral side.
Tarsus length	Length measured from the ankle to the proximal-most edge of the inner metatarsal tubercle while viewing the animal from the ventral side
Foot length	Length measured from the proximal-most edge of the inner metatarsal tubercle to the tip of the longest hind limb digit while viewing the animal from the ventral side
Front limb length	The sum of the upper front limb, forearm, and hand lengths
Hind limb length	The sum of the thigh, crus, tarsus, and foot lengths
Interdigital webbing area	The sum of area measurements between hind limb digits from photographs of outstretched digits pressed against a glass slide
Forelimb digit tip area	The sum of area measurements of forelimb digit tips from photographs of digit tips lightly pressed against a glass slide
Hindlimb Digit Tip Area	The sum of area measurements of hindlimb digit tips from photographs of digit tips lightly pressed against a glass slide
Inner metatarsal tubercle area	Area measurement of the inner metatarsal tubercle from photographs of the foot gently pressed against a glass slide
Thigh volume	Volume approximated as two cones sharing an elliptical base, pointed away from one another and meeting at the mid-point of the thigh. Lengths of the major and minor axes of the ellipse are the depth and width of the thigh at the mid-point, measured perpendicular to one another, which measurement is considered major or minor is arbitrary. The area of an ellipse is given by $A =$

	πab, where a and b are the semi-axes of the ellipse. The volume of a cone with an elliptical base is given by $V = HA/3$, where H is the height of the cone and A is the area of the ellipse. Since we approximate the volume of the entire thigh as two cones sharing the same base, we can sum their heights as the entire leg segment length (i.e., thigh) to calculate the volume of both cones together.
Crus volume	Similar to thigh volume but using crus measurements.

All lengths in mm, areas in mm^2, and volumes in mm^3.

Supplementary Table 5 | Effects of morphospace dimensionality on percentage of overall frog morphological volume occupied by each quadrant across the radiation space.

Convex hull					
4 Dimensions		5 Dimensions		6 Dimensions	
Quadrant	% Volume	Quadrant	% Volume	Quadrant	% Volume
AR	75.69%	AR	66.63%	AR	51.28%
ANR	21.06%	ANR	11.84%	ANR	8.16%
NANR	5.82%	NANR	2.38%	NANR	1.02%
NAR	26.75%	NAR	13.49%	NAR	6.54%

Hypervolume					
4 Dimensions		5 Dimensions		6 Dimensions	
Quadrant	% Volume	Quadrant	% Volume	Quadrant	% Volume
AR	74.69%	AR	75.43%	AR	73.92%
ANR	24.54%	ANR	15.48%	ANR	12.38%
NANR	5.2%	NANR	2.58%	NANR	1.5%
NAR	26.36%	NAR	14.13%	NAR	9.39%

Quadrant bounds are defined by the means of net diversification rates (stem ages, $\varepsilon=0.5$) and rates of multivariate morphological evolution. 'AR' is adaptive radiation; 'ANR' is adaptive, non-radiation; 'NANR' is non-adaptive, non-radiation; and 'NAR' is non-adaptive radiation. Percentages across quadrants may not sum to 100% because each quadrant's morphospace may variably overlap with other quadrant morphospaces. Overlap is higher when spaces are defined by fewer dimensions, generally reducing each quadrant's percentage as dimensionality increases. Source Data can be found within Supplementary Code 1.

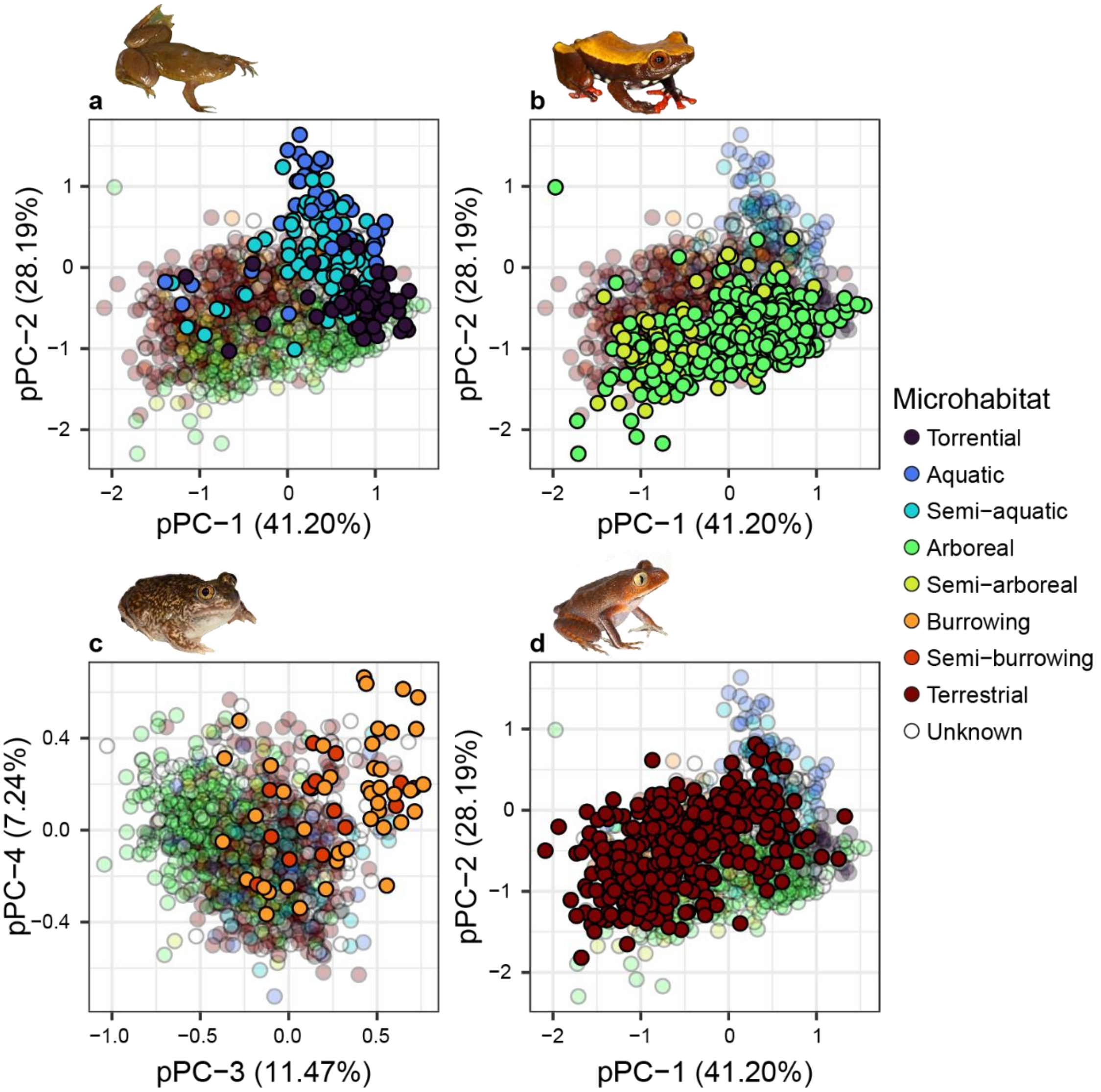

Supplementary Fig. 1 | Morphospaces characterized by pPC-1 and 2 (a, b, d), and pPC-3 and 4 (c) across 1,234 species. Each panel highlights a set of different microhabitats: (a) torrential, aquatic, and semi-aquatic (*n*=51, 40, and 90 species); (**b**) arboreal and semi-arboreal (*n*=315 and 91); (**c**) burrowing and semi-burrowing (*n*=43 and 18); (**d**) terrestrial (*n*=376). Photos represent the microhabitat in each panel: (**a**) *Xenopus tropicalis* (aquatic); (**b**) *Hyperolius bolifambae* (arboreal); (**c**) *Spea bombrifons* (burrowing); and (**d**) *Nyctibates corrugatus* (terrestrial). All photos by D.S.M. Source Data can be found within Supplementary Code 1.

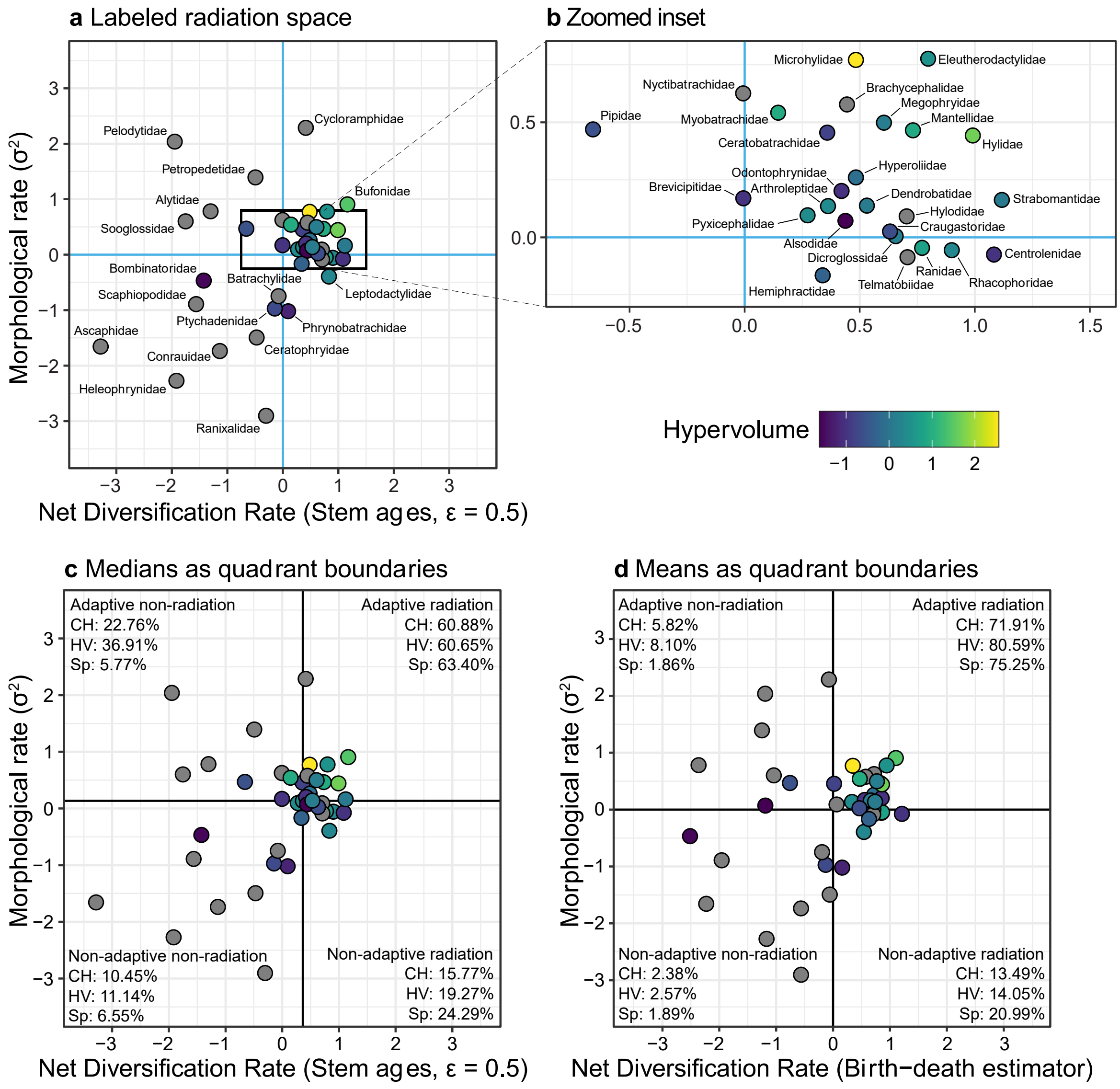

Supplementary Fig. 2 | Labeled radiation space (a,b) and two variations of the space in which quadrants are defined by rate medians (c) or net diversification rates are based on birth-death estimates (d). Scatterplots represent logged, mean-centered, and scaled rates of net diversification and multivariate morphological evolution for frog families (*n*=43). Percentages indicate the total species diversity (Sp) and five-dimensional morphological convex hull (CH) and hypervolume (HV) that each quadrant occupies. Panel (**b**) is an enlarged inset of panel (**a**) to more easily distinguish points; both are labeled copies of Fig. 4, with quadrant boundaries in blue to more easily read family names. Gray dots are families whose volumes could not be estimated. Source Data can be found within Supplementary Code 1.

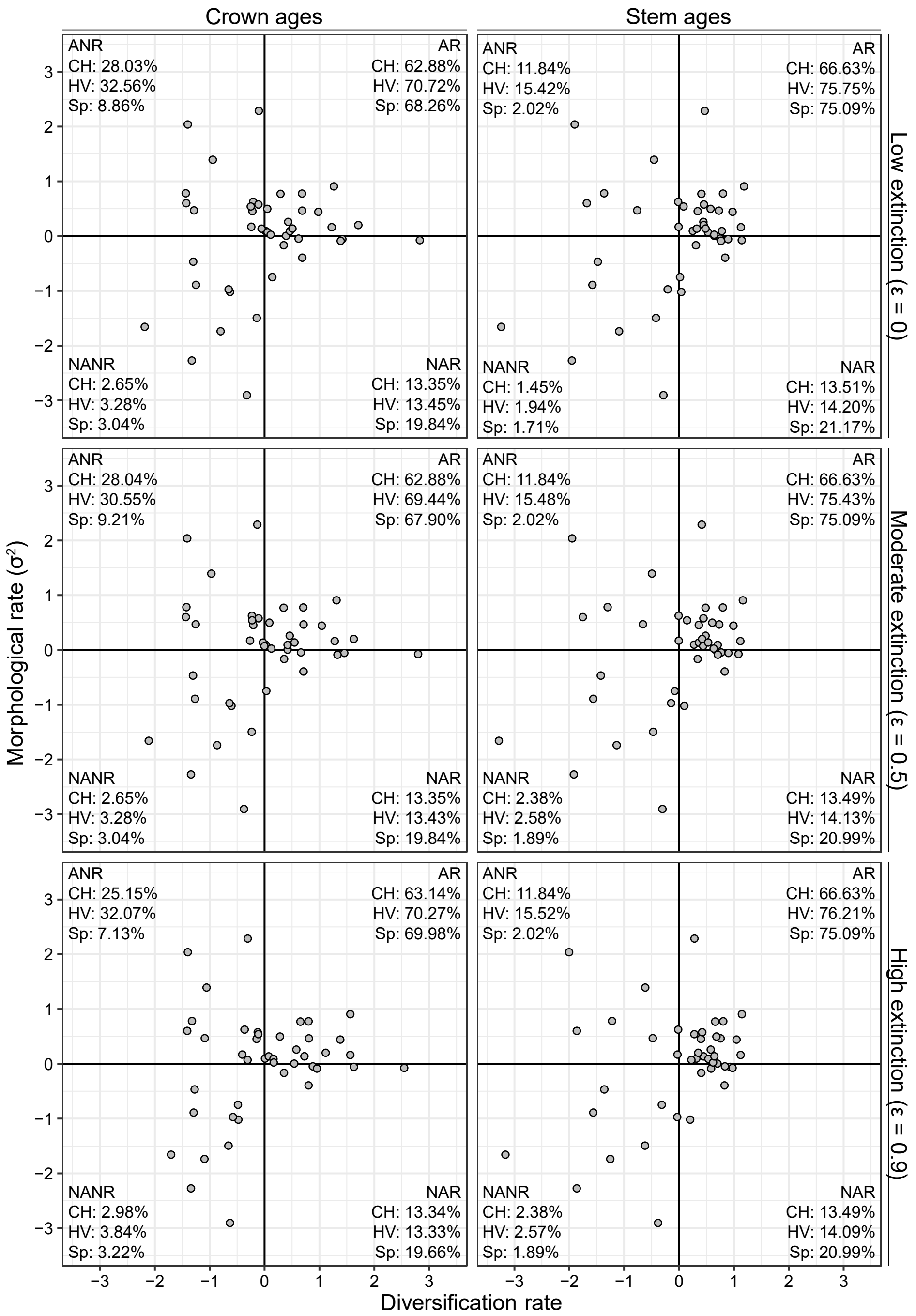

Supplementary Fig. 3 | Effect on the radiation space of different ways of calculating family-level diversification rates. Scatterplots represent logged, mean-centered, and scaled rates of net diversification and multivariate morphological evolution for frog families (*n*=43). Panels show results for diversification rates based on crown (left column) and stem (right column) ages, as well as differing extinction fractions (variation across rows). Quadrants are bounded by means. Acronyms, as well as all other details, follow Supplementary Fig. 2. Source Data can be found within Supplementary Code 1.

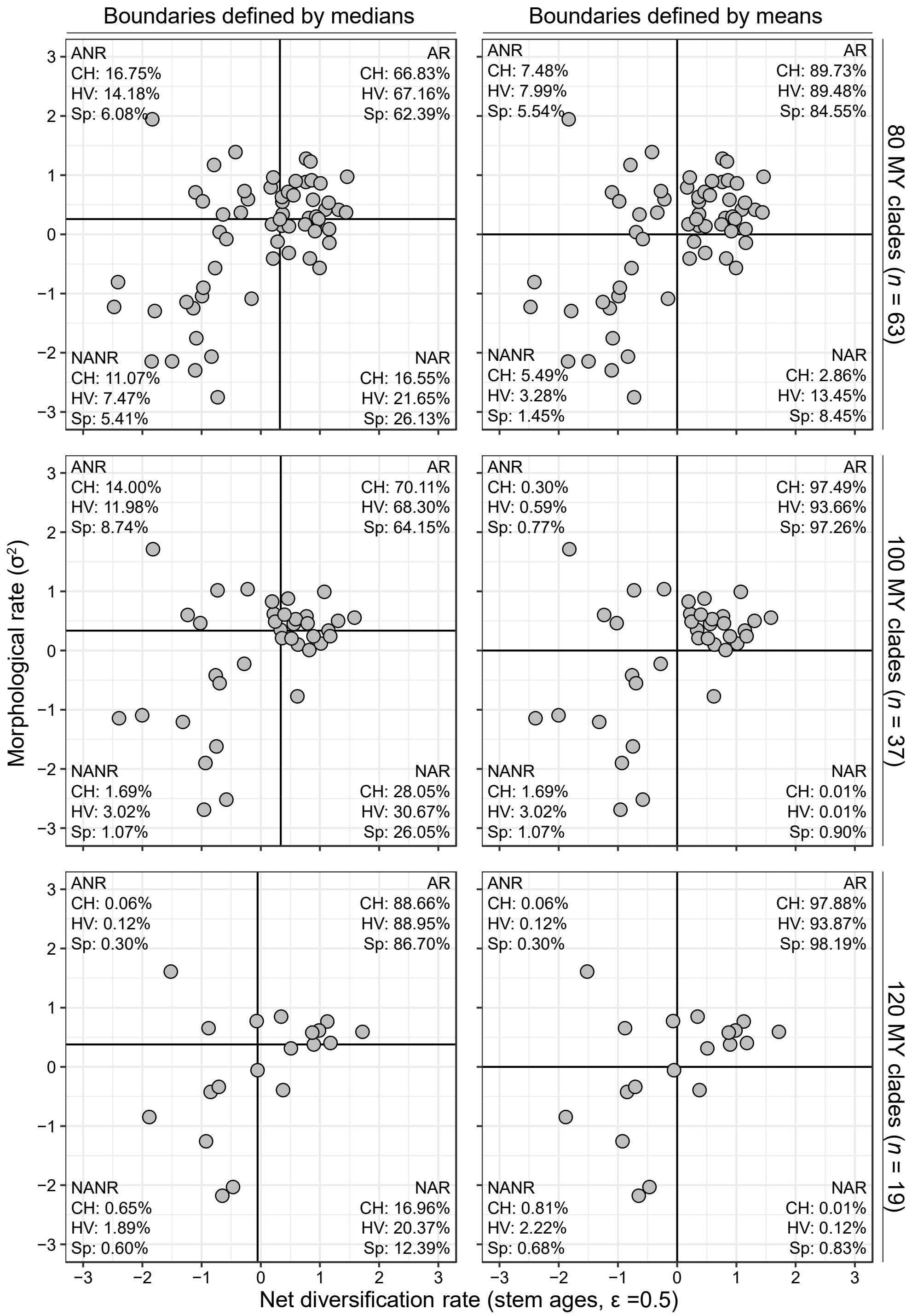

Supplementary Fig. 4 | Effect on the radiation space of defining clades by age, rather than family names. Scatterplots represent logged, mean-centered, and scaled rates of net diversification and multivariate morphological evolution. Grey dots represent clades; rows differ by the age that defines clades. Quadrants are delimited by either rate means (left column of panels) or medians (right column). All other details follow Supplementary Fig. 2. Source Data can be found within Supplementary Code 1.

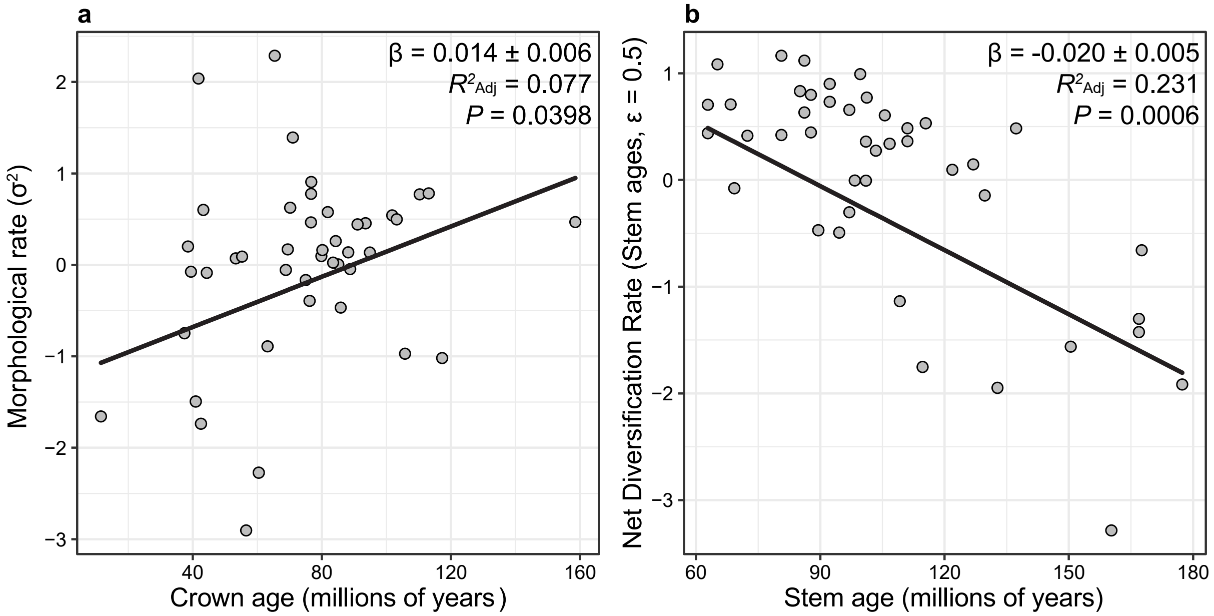

Supplementary Fig. 5 | Phylogenetic generalized least-squares regressions of rates of (a) multivariate morphological evolution and (b) net diversification on time. Grey dots represent rates for families (*n*=43) that were logged, mean-centered, and scaled. Morphological rates were regressed on crown age because only crown-level, family-level phylogenies are used to estimate rates. Net diversification rates were regressed on stem age because that was the age upon which these rates were based. *P*-values reflect two-tailed significance tests based on the regression slope. Source Data can be found within Supplementary Code 1.

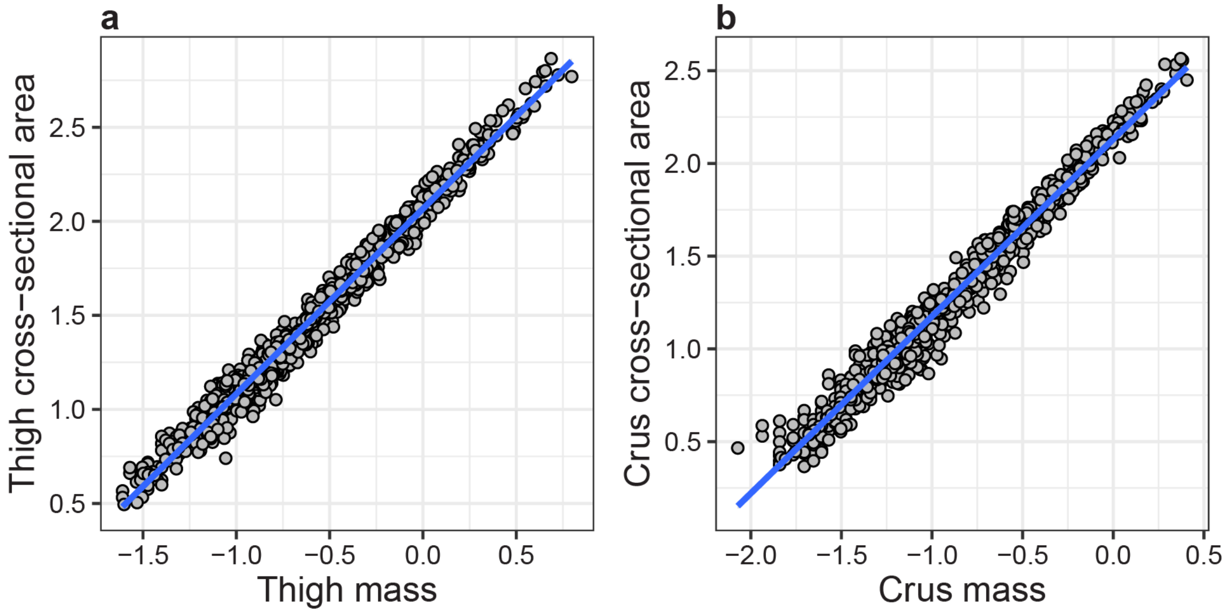

Supplementary Fig. 6 | Relationship between thigh (a) and crus (b) muscle mass and cross-sectional areas (CSA) for 641 specimens from 132 species. We square-rooted (CSA in mm^2) and cube-rooted (mass in grams) these measurements, then ln-transformed the resulting values. Blue line is the ordinary least squares (OLS) regression line. Gray dots represent individual frogs, with measurements for both muscle mass, and width and depth measures, of the thigh and crus. For 238 specimens across 49 species, we lacked thigh and crus width and depth measurements, but had mass measurements. These strong relationships (R^2=0.944 and 0.929 for thigh and crus, respectively) showed that we could use muscle mass to estimate CSA in these species, given our decision to analyze estimated muscle volume (i.e., we had many more specimens for which we had only CSA measurements: 4,073 specimens from 1,071 species). Thus, we used the OLS regression to predict thigh and crus CSA for these specimens from their masses. Source Data can be found within Supplementary Code 1.

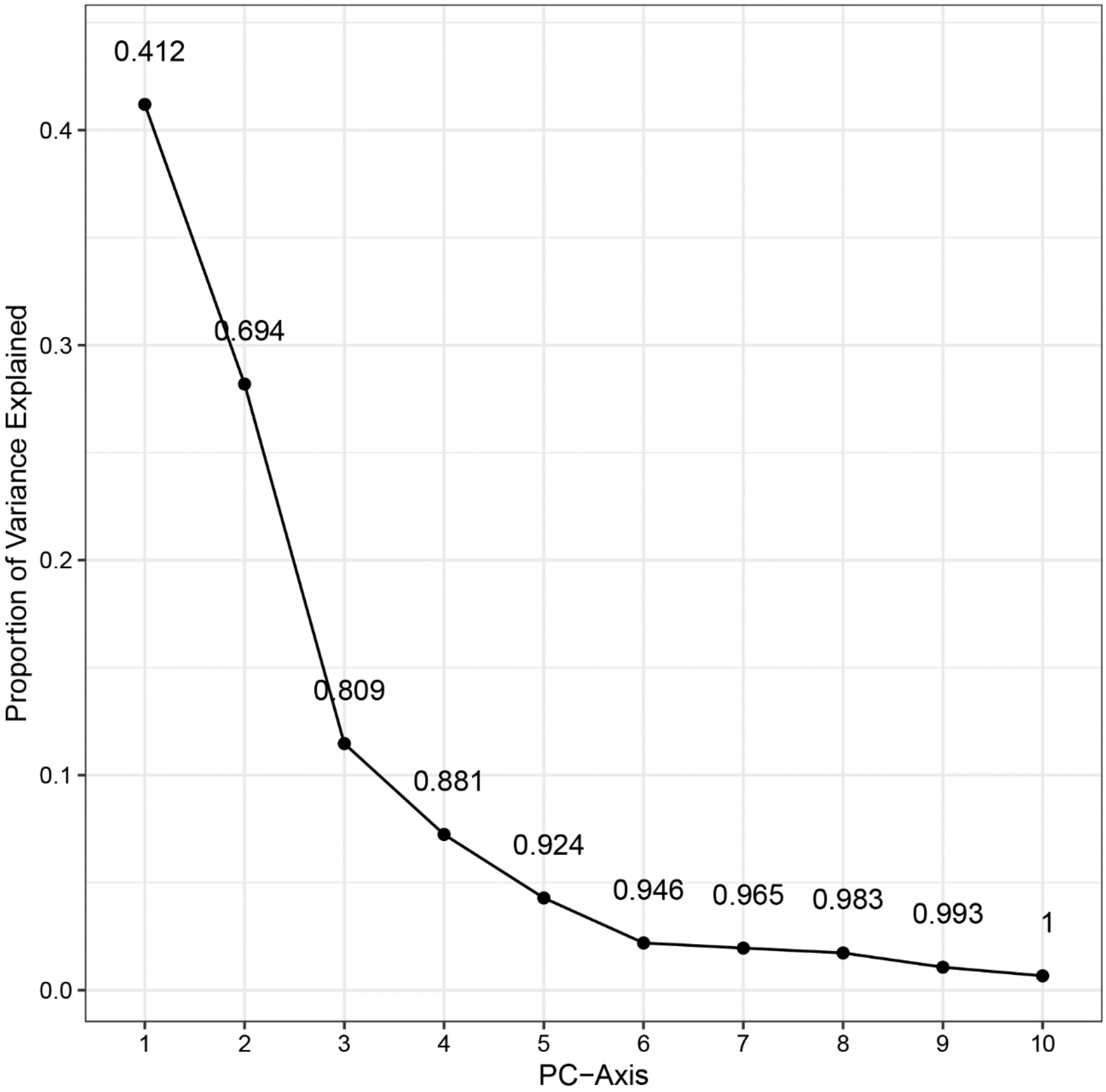

Supplementary Fig. 7 | Scree plot showing the variance explained by each axis of our phylogenetic principal components analysis. Height of the black dots show the proportion of variance explained by each pPC axis. The numbers above each dot show the cumulative variance explained all that axis and all lower axes combined. The line connecting each dot illustrates the drop in variance explained. Source Data can be found within Supplementary Code 1.
