# Supplementary material for: The radiation continuum and the evolution of frog diversity
Source: Nat Commun. 2023 Nov 4;14:7100. doi: 10.1038/s41467-023-42745-x (PMC10625520; doi:10.1038/s41467-023-42745-x)
Supplement: Supplementary file 3 — Description of Additional Supplementary Files [file 41467_2023_42745_MOESM3_ESM.pdf]

Description of Additional Supplementary File for

# The radiation continuum and the evolution of frog diversity

Gen Morinaga[1,2], John J. Wiens[3], and Daniel S. Moen[1,4,*]

[1]Department of Integrative Biology, Oklahoma State University, Stillwater, OK 74078, USA. [2]Department of Comparative Biology, University of Calgary, Calgary, Alberta T2N 4N1, Canada. [3]Department of Ecology and Evolutionary Biology, University of Arizona, Tucson, AZ 85721, USA. [4]Department of Evolution, Ecology, and Organismal Biology, University of California, Riverside, Riverside, CA 92521, USA.

*Corresponding author: dmoen@ucr.edu

## Data files

"Supplementary Data 1": The full intraspecific phenotypic dataset. The vast majority of these data are raw measurements. The infrequent values with more than three digits were estimated based on intraspecific scaling relationships, due to unavailability of photos. Provided as an MS Excel file with a tab of metadata.

"Supplementary Data 2": Interspecific species means, standard deviations, standard errors calculated per species, and standard errors calculated with a pooled estimate of measurement variance from Ives *et al.* (2007). All values were rounded to three digits; wherever fewer digits occur, these values rounded to end in one or more zeros. Provided as an MS Excel file with a tab of metadata.

"Supplementary Data 3": Full documentation of our microhabitat classifications for taxa new to this study (i.e., not coming from Moen and Wiens [2017]). The file includes a data tab with short reference information and a reference tab with the full references.

"Supplementary Data 4": Ages, species diversity, and raw rates of morphological evolution and net diversification estimated for each family in this paper. These are the rates most amenable to comparison with other studies.

"Supplementary Data 5": Ages, species diversity, and transformed (logged, centered, and scaled) rates of morphological evolution and net diversification estimated for each family in this paper. These were the rates we used for most analyses.

## Data analysis documentation, zipped as Supplementary Code 1

"amph_shl_new_Consensus_7238.tre": Consensus of 10,000 fully-sampled trees from the posterior distribution of Jetz and Pyron (2018), containing species with genetic data

(4,061) and imputed taxa (3,177). We used this tree for estimating species diversity of time-sliced clades.

"aw.latest.csv": A file from AmphibiaWeb (2021) that we used to calculate current species diversity of each anuran family. This was the diversity on AmphibiaWeb on the date of our accession for these analyses, 7 December 2021.

"JP_NatEE_2018_3349tax_ultra.nex": A nexus file of the ultrametric maximum-clade credibility tree of the anuran taxa for which Jetz and Pyron (2018) had genetic data. This tree was the basis for most of our analyses.

"Moen_etal_2021_SuppInfoS9.diversification.data.csv": We used this file from Moen *et al.* (2021) to extract birth-death net diversification-rate estimates for anuran families.

"Morphometrics_anura_4628_specimens.csv": The full intraspecific phenotypic dataset. This file is the same as Supplementary Data 4 (see above for details) but renamed here for using in the analysis.

"Radiation_continuum_essential_objects.RData": This R data file is loaded for facilitating data analysis in our R Markdown document.

"Radiation_continuum": An R Markdown tutorial that walks readers through our analyses. We provide the optimal viewing format (HTML file), in addition to the raw R Markdown (.Rmd) and just the R code (.R). The latter two files facilitate quick analysis and review of code.

**References**

AmphibiaWeb. AmphibiaWeb: Information on amphibian biology and conservation, http://amphibiaweb.org (2021). Accessed 7 December 2021.

Ives, A. R., P. E. Midford, & T. Garland, Jr. Within-species variation and measurement error in phylogenetic comparative methods. *Syst. Biol.* 56, 252–270 (2007).

Jetz, W. & Pyron, R. A. The interplay of past diversification and evolutionary isolation with present imperilment across the amphibian tree of life. *Nat. Ecol. Evol.* 2, 850–858 (2018).

Moen, D. S., & J. J. Wiens. Microhabitat and climatic niche change explain patterns of diversification among frog families. *Am. Nat.* 190, 29–44 (2017).

Moen, D. S., Ravelojaona, R. N., Hutter, C. R. & Wiens, J. J. Testing for adaptive radiation: A new approach applied to Madagascar frogs. *Evolution* 75, 3008–3025 (2021).
