# Supplementary material for: The radiation continuum and the evolution of frog diversity
Source: Nat Commun. 2023 Nov 4;14:7100. doi: 10.1038/s41467-023-42745-x (PMC10625520; doi:10.1038/s41467-023-42745-x)
Supplement: Supplementary file 9 — Supplementary Code 1 [file 41467_2023_42745_MOESM9_ESM.zip › Supplementary Code 1/Radiation_continuum.html]

Detailed analysis methods for: The radiation continuum and the evolution of frog diversity


# Detailed analysis methods for: The radiation continuum and the evolution of frog diversity

#### Gen Morinaga, John J. Wiens, and Daniel S. Moen

#### 10 October 2023

In this document we provide all the R code we used to produce the
results, figures, and tables from the accompanying manuscript published
in *Nature Communications*. While we provide some key references,
full method justification and references can be found in the published
paper. For simpler replication of these analyses in R, we recommend
using the .R or .Rmd files associated with this HTML document. They work
best in RStudio.

First, we list the packages required to perform all analyses in this
document. We used R version 4.1.0.

```
library(tidyr) #version 1.2.0
library(dplyr) #version 1.0.6
library(geiger) #version 2.0.7
library(geomorph) #version 4.0.1
library(phytools) #version 1.0.1
library(ape) #version 5.5
library(vegan) #version 2.5.7
library(ggplot2) #version 3.3.5
library(geometry) #version 0.4.5
library(hypervolume) #version 3.0.2
library(patchwork) #version 1.1.1
library(viridis) #version 0.6.2
library(scales) #version 1.1.1
library(hexbin) #version 1.28.2
library(ggnewscale) #version 0.4.5
library(tibble) #version 3.1.2
library(ggrepel) #version 0.9.1
library(cowplot) #version 1.1.1
library(phylolm) #version 2.6.2

####ggtree is not available on CRAN. To install this package use the following code:
# if (!require("BiocManager", quietly = TRUE))
#     install.packages("BiocManager")
# 
# BiocManager::install("ggtree")

library(ggtree) #version 3.0.4
```

## Loading the workspace and preparing data for analysis

```
### Load the RData file necessary to recreate these analyses. This should be in your active working directory.
load('./Radiation_continuum_essential_objects.RData') 
ls()
```

```
##  [1] "aw.latest"            "bd.hv.table"          "bd.rates"            
##  [4] "cor.pvalue"           "dat"                  "dat.100.hv.table"    
##  [7] "dat.100.med.hv.table" "dat.120.hv.table"     "dat.120.med.hv.table"
## [10] "dat.80.hv.table"      "dat.80.med.hv.table"  "get.vol"             
## [13] "get.vol.4d"           "get.vol.6d"           "gls.corr"            
## [16] "jp.tree"              "jp7k"                 "quads.4d.hv.table"   
## [19] "quads.6d.hv.table"    "quads.hv.table"       "quads.med.hv.table"  
## [22] "vol.evo"
```

The necessary files to re-create these analyses are pre-loaded into
the RData file called ‘Radiation\_continuum\_essential\_objects.RData’.
This document will go through each step taken to process the data for
analyses and through the analyses proper. The objects `dat`,
`jp.tree`, `aw.latest`, `jp7k`,
`bd.rates` are particularly important for re-creating these
analyses, as they are essentially “raw” data. `dat` is the
morphological data from over 4600 frog specimens (the original file is
also included and called ‘Morphometrics\_anura\_4628\_specimens.csv’).
`jp.tree` is a consensus tree of 100 draws from vertlife.org,
originally published by Jetz & Pyron (2018; the original file is
included and called ‘JP\_NatEE\_2018\_3349tax\_ultra.nex’).
`jp7k` is from the same publication (in their supplementary
materials), but is the consensus tree after all named taxa without
genetic data are placed onto the backbone of the original tree published
by Jetz & Pyron (2018; the original file is included and called
‘amph\_shl\_new\_Consensus\_7238.tre’). `aw.latest` is the csv
output of amphibian taxonomy from amphibiaweb.org, which we retrieved on
Dec-08-2021 (we included this as ‘aw.latest.csv’). `bd.rates`
is a data set published by Moen et al. (2021), included as
‘Moen\_etal\_2021\_SuppInfoS9.diversification.data.csv’.

Additionally, we load several custom functions onto the workspace
that are either from other publications or are very long.
`gls.corr` and `cor.pvalue` are functions used to
estimate pGLS correlations originally written and used in Moen et
al. (2013). `get.vol` is a custom written wrapper function
for `geometry::convhulln` and
`hypervolume::hypervolume_svm` to estimate convex hulls and
hypervolumes for different quadrants of the radiation space.

Lastly, hypervolume objects are time consuming to estimate both due
to the large size of our dataset and the stochastic nature of the
estimation process. For this reason, we separately provide all
hypervolume objects created for these analyses in a the
‘frog\_hypervolumes.RData’ file. Here, we have loaded the tables derived
from each of the hypervolume objects, but provide code (commented out in
the code fields) that can be used estimate and re-create the tables we
used below. These objects all end with `.table`, with the
exception of `vol.evo`.

### Preparing the phylogeny

Three taxa have taxon labels that do not match those on AmphibiaWeb.
We corrected this by changing them to AmphibiWeb’s taxonomy.

```
jp.tree$tip.label[c(775, 776, 822)]<- c('Sallywalkerana_diplosticta', 'Sallywalkerana_leptodactyla', 'Alcalus_baluensis')
```

A quick inspection of the plot for `jp.tree` shows it is
already ultrametric. Sometimes such trees fail a formal test of being
ultrametric, a key (internal) step of many downstream analyses.

```
plot(jp.tree, show.tip.label = F)
```

```
is.ultrametric(jp.tree)
```

```
## [1] TRUE
```

When this test gives `FALSE` but a plot shows an
ultrametric tree, rounding errors from numerical precision in R are the
source of the discordance. We will fix this error by forcing using
`force.ultrametric`. Note that this is not an appropriate way
to scale non-ultrametric branches (e.g., when converting a phylogeny
with uneven genetic branch lengths into an ultrametric tree).

```
jp.tree<-force.ultrametric(jp.tree)
```

```
## ***************************************************************
## *                          Note:                              *
## *    force.ultrametric does not include a formal method to    *
## *    ultrametricize a tree & should only be used to coerce    *
## *   a phylogeny that fails is.ultramtric due to rounding --   *
## *    not as a substitute for formal rate-smoothing methods.   *
## ***************************************************************
```

```
is.ultrametric(jp.tree)
```

```
## [1] TRUE
```

Later we will prune the tree to only include taxa in our
morphological dataset.

### Preparing the morphometric dataset

#### Approximating volumes of the thigh and crus

We approximated volumes of the thigh and crus by generalizing each as
a pair of cones with an elliptical cross section, with both cones
connected at the base and pointing away from one another (Juarez and
Adams 2022). Recall that the area of an ellipse is given by \(A\_{ellipse} = \pi ab\), where \(a\) and \(b\) are the major and minor semi-axes of an
ellipse, which we substituted with thigh (or crus) width and depth
measurements each multiplied by 0.5 (because *half* of each
measurement would constitute a semi-axis). Next recall that the volume
of a cone is given by \(V\_{cone} =
\frac{1}{3}\pi r^{2}h\). We can replace the circular base for an
elliptical one. Rather than \(\pi
r^{2}\) (the area of a circle), substitute \(\pi ab\). Additionally, \(h\) is height of the cone. For just one
cone, \(h\) would be half of the thigh
(or crus) length. Since we approximated the thigh (or crus) volume as
*two* cones, we simply used the full length of the thigh (or
crus).

Substituting our thigh or crus measurements into the formula yields
\(V = \frac{1}{3}\pi \frac{width}{2}
\frac{depth}{2} length\). For most specimens in this dataset, we
could directly estimate thigh and crus volumes because we measured
width, depth, and length directly. However, for a subset of specimens,
width and depth measurements were not taken. Instead, thigh and crus
mass were measured. Another subset of specimens have all thigh and crus
measurements (i.e., length, width, depth, and mass). To maximize the
number of specimens (and thus species) we could include, we took
advantage of these latter specimens to extrapolate \(V\) from mass.

We started by isolating specimens that had thigh and crus muscle
masses and calculated cross-sectional areas (ellipse) from those that
also had widths and depths.

```
est.csa <- dat %>%
  select(specimen, species, family, microhabitat, svl:hand.pad) %>%
  filter(!is.na(thigh.mass)) %>% 
  mutate(thigh.csa = (thigh.D/2)*(thigh.W/2)*pi, 
         crus.csa = (crus.D/2)*(crus.W/2)*pi)
```

We next tested how strong the cross-sectional areas and masses were
correlated with one another. Finding very high correlations, we then
fitted a linear model (ordinary least-squares) of the ln-transformed
thigh and crus CSA against ln-transformed thigh and crus muscle mass.
Note that areas were square-rooted and masses were cube-rooted for these
analyses.

```
with(est.csa, cor.test(log(sqrt(thigh.csa)), log(thigh.mass^(1/3))))
```

```
## 
##  Pearson's product-moment correlation
## 
## data:  log(sqrt(thigh.csa)) and log(thigh.mass^(1/3))
## t = 106.44, df = 593, p-value < 2.2e-16
## alternative hypothesis: true correlation is not equal to 0
## 95 percent confidence interval:
##  0.9704736 0.9785195
## sample estimates:
##       cor 
## 0.9748118
```

```
with(est.csa, cor.test(log(sqrt(crus.csa)), log(crus.mass^(1/3))))
```

```
## 
##  Pearson's product-moment correlation
## 
## data:  log(sqrt(crus.csa)) and log(crus.mass^(1/3))
## t = 92.393, df = 593, p-value < 2.2e-16
## alternative hypothesis: true correlation is not equal to 0
## 95 percent confidence interval:
##  0.9613168 0.9718223
## sample estimates:
##       cor 
## 0.9669778
```

```
thigh.fit <- lm(log(sqrt(thigh.csa)) ~ log(thigh.mass^(1/3)), data = est.csa)
crus.fit <-lm(log(sqrt(crus.csa)) ~ log(crus.mass^(1/3)), data = est.csa)
summary(thigh.fit)
```

```
## 
## Call:
## lm(formula = log(sqrt(thigh.csa)) ~ log(thigh.mass^(1/3)), data = est.csa)
## 
## Residuals:
##      Min       1Q   Median       3Q      Max 
## -0.38350 -0.08379 -0.01535  0.07909  0.37128 
## 
## Coefficients:
##                       Estimate Std. Error t value Pr(>|t|)    
## (Intercept)           1.950763   0.008151   239.3   <2e-16 ***
## log(thigh.mass^(1/3)) 1.065334   0.010009   106.4   <2e-16 ***
## ---
## Signif. codes:  0 '***' 0.001 '**' 0.01 '*' 0.05 '.' 0.1 ' ' 1
## 
## Residual standard error: 0.1244 on 593 degrees of freedom
##   (229 observations deleted due to missingness)
## Multiple R-squared:  0.9503, Adjusted R-squared:  0.9502 
## F-statistic: 1.133e+04 on 1 and 593 DF,  p-value: < 2.2e-16
```

```
summary(crus.fit)
```

```
## 
## Call:
## lm(formula = log(sqrt(crus.csa)) ~ log(crus.mass^(1/3)), data = est.csa)
## 
## Residuals:
##      Min       1Q   Median       3Q      Max 
## -0.36978 -0.09137 -0.01052  0.09132  0.37016 
## 
## Coefficients:
##                      Estimate Std. Error t value Pr(>|t|)    
## (Intercept)           1.89113    0.01149  164.57   <2e-16 ***
## log(crus.mass^(1/3))  1.01373    0.01097   92.39   <2e-16 ***
## ---
## Signif. codes:  0 '***' 0.001 '**' 0.01 '*' 0.05 '.' 0.1 ' ' 1
## 
## Residual standard error: 0.1308 on 593 degrees of freedom
##   (229 observations deleted due to missingness)
## Multiple R-squared:  0.935,  Adjusted R-squared:  0.9349 
## F-statistic:  8537 on 1 and 593 DF,  p-value: < 2.2e-16
```

We then used these models to extrapolate a CSA for the thigh and crus
from their respective masses. Keep in mind that the CSAs that went into
the original models were ln-transformed and square-rooted; thus, the
predicted values from these models must be exponentiated and squared.
Using the extrapolated CSAs, we estimated volumes according the formula
above.

```
#Estimate CSA
est.csa$thigh.csa.pred <-exp(predict(thigh.fit, newdata = data.frame(thigh.mass = est.csa$thigh.mass)))^2
est.csa$crus.csa.pred <-exp(predict(crus.fit, newdata = data.frame(crus.mass = est.csa$crus.mass)))^2
#Estimate volumes from CSA
to.dat <- est.csa %>%
  filter(is.na(thigh.D)) %>%
  select(-thigh.csa, -crus.csa) %>%
  mutate(thigh.v = thigh.csa.pred*thigh.L/3, crus.v = crus.csa.pred*crus.L/3)
```

We validated our extrapolated muscle volumes by checking whether they
scaled with mass at approximately 1:1, as seen on Supplementary Figure
6.

```
vol.check<-est.csa %>%
  filter(!is.na(thigh.D)) %>%
  mutate(thigh.v = (1/3 * ((thigh.D/2) * (thigh.W/2) * pi) * thigh.L),
         crus.v = (1/3 * ((crus.D/2) * (crus.W/2) * pi) * crus.L)) %>%
  bind_rows(., to.dat)

#Fit linear models
(lm(log(thigh.v^(1/3))~log(thigh.mass^(1/3)), data=vol.check))
```

```
## 
## Call:
## lm(formula = log(thigh.v^(1/3)) ~ log(thigh.mass^(1/3)), data = vol.check)
## 
## Coefficients:
##           (Intercept)  log(thigh.mass^(1/3))  
##                2.0669                 0.9844
```

```
(lm(log(crus.v^(1/3))~log(crus.mass^(1/3)), data=vol.check))
```

```
## 
## Call:
## lm(formula = log(crus.v^(1/3)) ~ log(crus.mass^(1/3)), data = vol.check)
## 
## Coefficients:
##          (Intercept)  log(crus.mass^(1/3))  
##                2.131                 0.955
```

```
#Check visually, plot regression lines
(thigh.crus.plot<-ggplot(vol.check, aes(x = log(thigh.mass^(1/3)), y = log(thigh.v^(1/3)))) +
  geom_point(shape = 21, fill = 'grey') +
  stat_smooth(method = 'lm') + 
  scale_x_continuous(breaks = pretty_breaks()) +
  scale_y_continuous(breaks = pretty_breaks()) +
  xlab('Thigh mass') +
  ylab('Thigh cross-sectional area') +
  ggtitle('a.') +
  theme_bw() +
  theme(aspect.ratio = 1) +
  ggplot(vol.check, aes(x = log((crus.mass)^(1/3)), log(crus.v^(1/3)))) +
  geom_point(shape = 21, fill = 'grey') +
  stat_smooth(method = 'lm') + 
  xlab('Crus mass') +
  ylab('Cruss cross-sectional area') +
  ggtitle('b.') +
  theme_bw() +
  theme(aspect.ratio = 1))
```

```
## `geom_smooth()` using formula = 'y ~ x'
## `geom_smooth()` using formula = 'y ~ x'
```

After validating that mass was a good predictor of volume, we
combined the estimated volumes with the rest of the dataset.

```
dat2<-dat %>%
  filter(!is.na(thigh.D)) %>%
  select(specimen:hand.pad) %>%
  mutate(thigh.v = (1 / 3 * ((thigh.D/2) * (thigh.W/2) * pi) * thigh.L),
         crus.v = (1 / 3 * ((crus.D/2) * (crus.W/2) * pi) * crus.L)) %>%
  bind_rows(., to.dat)
```

#### Estimating species means and size-correction

##### Size-correction using ratios

`dat2` now contains all of the relevant measurements, but
we still needed to combine some measurements and calculate species
means. We first combined individual limb element measurements to produce
front and hind limb length. We then estimated species means. Then, we
size-corrected all measurements by dividing each by SVL. Webbing,
adhesive pad, and inner metatarsal tubercles measurements were areas, so
they needed to be square-rooted prior to size-correction. Similarly,
volume measurements needed to be cube-rooted prior to size-correction.
All measurements were subsequently ln-transformed.

```
mean.ratio.dat<-dat2 %>%
mutate(hind.limb.L = thigh.L + crus.L + tarsus.L + foot.L, front.limb.L = hand.L + forearm.L + upper.arm.L) %>%
       select(specimen:microhabitat, svl, head.L,head.W, front.limb.L, hind.limb.L, foot.web, foot.pad, hand.pad, inner.mtt, thigh.v, crus.v) %>%
       #calculate means
       group_by(species, family, microhabitat) %>% 
       summarise(across(svl:crus.v, \(x) mean(x, na.rm =T))) %>%
       #size-correct by dividing each variable by svl and ln transform
       mutate(across(head.L:hind.limb.L, ~ log(.x/svl))) %>% #simply divide linear measurements then ln transform
       mutate(across(foot.web:inner.mtt, ~ log(sqrt(.x)/svl))) %>% #square-root area measurements then ln transform
       mutate(across(thigh.v:crus.v, ~ log(.x^(1/3)/svl))) %>% #cube-root volume measurement then ln transform
       select(-svl) %>% #svl is no longer necessary, so we will remove it
       data.frame(.)
```

```
## `summarise()` has grouped output by 'species', 'family'. You can override using
## the `.groups` argument.
```

Lastly, since we had a finalized dataset of size-corrected species
means, we pruned `jp.tree` to includ only taxa that occurred
in our dataset. Normally, `treedata` will warn users of the
taxa getting excluded. We have turned off the warnings here to save
space.

```
sp.tree<-treedata(jp.tree, mean.ratio.dat %>% column_to_rownames('species'), sort = T, warnings = F)$phy
```

##### Size-correction using residuals

Another way that size-correction can be achieved is by taking the
residuals from the regression of the variables of interest against the
size variable. These methods ultimately yielded similar intermediate
results (see below). However, we note that a number of authors caution
against the treatment of residuals as data. For this reason, we did not
use residual size-corrected data for our final analyses.

```
###Prepare species means for phyl.resid
for.resid.dat<-dat2 %>%
  mutate(hind.limb.L = thigh.L + crus.L + tarsus.L + foot.L,
         front.limb.L = hand.L + forearm.L + upper.arm.L) %>%
  select(specimen:microhabitat, svl, head.L, head.W, front.limb.L,  hind.limb.L, foot.web, foot.pad, hand.pad, inner.mtt, thigh.v, crus.v) %>%
  mutate(across(foot.web:inner.mtt, ~ sqrt(.x)),
         across(thigh.v:crus.v, ~ (.x)^(1/3))) %>%
  group_by(species, family) %>%
  summarise(across(svl:crus.v, ~ mean(.x))) %>%
  mutate(across(svl:crus.v, ~ log(.x))) %>%
  column_to_rownames('species')
```

```
## `summarise()` has grouped output by 'species'. You can override using the
## `.groups` argument.
```

```
###set the row order to match the order of the tree
for.resid.dat<-data.frame(treedata(sp.tree, for.resid.dat %>% select(svl:crus.v), sort = T)$data)

###Get size-corrected residual trait values
mean.resid.dat<- data.frame(phyl.resid(tree = sp.tree, x = for.resid.dat %>% rownames_to_column('species') %>% pull(svl, name = species), Y = for.resid.dat %>% select(head.L:crus.v), method = 'BM')$resid)
```

## Estimating rates of multivariate morphological evolution

We estimated multivariate rates of morphological evolution for each
family in our dataset using `geomorph::compare.evol.rates`
(Adams 2014). To do this analysis, we created a list of subsets of
`mean.ratio.dat`, where each list item contained data from a
single family. We created a list of trees that matched the taxon
sampling in each listed subset. Note that some families have *n*
= 1 and so evolutionary rates cannot be estimated. We omitted those
eight families from this analysis.

```
###create a list of subsetted dataframes
mean.ratio.dat$family<-as.factor(mean.ratio.dat$family)
mean.ratio.dat.list<-lapply(levels(mean.ratio.dat$family), function (x) 
  mean.ratio.dat %>%
    select(species, family, head.L:crus.v) %>%
    column_to_rownames('species') %>%
    filter(family == x))
names(mean.ratio.dat.list)<-levels(mean.ratio.dat$family)
#remove families where n = 1
mean.ratio.dat.list<-mean.ratio.dat.list[sapply(mean.ratio.dat.list, nrow) > 1]
####create a list of tree that match the species represented in each dataframe in the list
sp.tree.list<-lapply(mean.ratio.dat.list, function(x) treedata(sp.tree, x, sort = T, warnings = F)$phy)
```

`compare.evol.rates` requires a dataframe or matrix, a
phylogeny, and a vector of groupings. Since we did not compare groups,
we simply parsed a named vector of 1s. The function also requires an
`iter` input for hypothesis testing. Since we simply used
`compare.evol.rates` to estimate evolutionary rates and not
to test any hypotheses, we set `iter = 1`.

```
#this is a simple function that creates a list of named vectors that contain 1s for each clade
gplist<-function(x) {
  lapply(x, function(x) {
    z<-rep(1, nrow(x))
    names(z)<-rownames(x)
    z
  })
}
gp.fam<-gplist(mean.ratio.dat.list)
#run compare.evol.rates on our list objects by using an mapply wrapper
fam.dat<-data.frame(ratio.sigma = mapply(
  function(x, y, z) {
    compare.evol.rates(A = select(x, -family), phy = y, gp = z, iter = 1)$sigma.d.all
  },
  x = mean.ratio.dat.list, y = sp.tree.list, z = gp.fam)) %>%
  rownames_to_column('family')
```

## Estimating net diversification rates

We primarily used the method-of-moments estimator introduced by
Magallón & Sanderson (2001) to estimate net diversification rates
for each family. We first needed to obtain species diversity for each
family. We used the taxonomy of AmphibiaWeb. New
taxa are added to this list nearly daily. So to recreate the species
diversity at the time our analyses, we used here the
`aw.latest` object loaded into the workspace (i.e., rather
than downloading the data from AmphibiaWeb).

```
#if the reader wants to retrieve the latest list, that can be done with the following code assuming they are connected to the internet:
# aw.latest<-read.csv('https://amphibiaweb.org/amphib_names.txt', sep = '\t')

#count the number of speces in each anuran family
aw.summarised <- aw.latest %>%
  filter(order == 'Anura') %>%
  group_by(family) %>%
  summarise(n = n())
```

The taxonomy list from AmphibiaWeb contains all amphibians. We subset
this list to include only anurans. Furthermore, the format of the
dataframe is a little inconvenient for our purposes, so we fixed that
here.

```
aw.taxa<-aw.latest %>%
  filter(order == 'Anura') %>%
  select(family, genus, species) %>%
  mutate(genus_species = paste(genus, species, sep = '_'))
rownames(aw.taxa) <- aw.taxa$genus_species
```

### Obtaining crown and stem age estimates

One component necessary for estimating diversification rates using
the method-of-moments estimator is clade age. Clade age will differ
based on whether we consider crown or stem ages. We used
`jp.tree` to obtain both crown and stem ages.

First we obtained crown ages by extracting a subtree of each family
and finding the maximum branching time of that subtree. Note that this
code will simply parse `NA` for families that were monotypic
or only had a single taxon sampled in the phylogeny.

```
jp.tree2<-treedata(jp.tree, aw.taxa)$phy
aw.jp.taxa<-data.frame(treedata(jp.tree2, aw.taxa, warnings = F)$data)
aw.jp.taxa$family<-as.factor(aw.jp.taxa$family)
fam<-levels(aw.jp.taxa$family)
fam.dat<-data.frame(family = fam,
                    crown.age = sapply(fam, function (x)
                      tryCatch({
                        max(branching.times(extract.clade(
                          jp.tree2,
                          findMRCA(jp.tree2,
                                   tip = aw.jp.taxa[which(aw.jp.taxa$family ==
                                                                 x),
                                                         'genus_species'])
                        )))
        ###because some families only have a single taxon in the tree (or are monotypic)
        ###this script will throw an error. To avoid this error, tryCatch() will feed
        ###NA for such families' crown age estimates
                      }, error = function(e)
                        NA))) %>%
  left_join(aw.summarised) %>%
  left_join(fam.dat)
```

```
## Joining with `by = join_by(family)`
## Joining with `by = join_by(family)`
```

Next we estimated stem ages. We took advantage of the fact that when
clades are pruned to include only a single species on a phylogeny, nodes
collapse to the tip and are added to the stem. Thus the edge length for
each terminal branch was the stem age.

```
#create a dataframe of consisting of taxon names of one species from each family
aw.tree.fam<-aw.jp.taxa %>%
  group_by(family) %>%
  slice_head(n = 1) %>%
  mutate(new = genus_species) %>%
  column_to_rownames('new')

#use the new dataframe to prune the tree down to just those present in the dataframe
fam.tree<-treedata(jp.tree2, aw.tree.fam, warnings = F)$phy

#match the order in the dataframe to match that of the new tree then change the tip labels to be family name
aw.tree.fam<-aw.tree.fam[match(fam.tree$tip.label, aw.tree.fam$genus_species),]
fam.tree$tip.label<-aw.tree.fam$family
fam.dat<-data.frame(stem.age = fam.tree$edge.length[fam.tree$edge[,2] <= Ntip(fam.tree)],
                    family = fam.tree$tip.label) %>%
  left_join(fam.dat) %>%
  relocate(family, .before = stem.age) %>%
  #simply drop the rows with NAs as they won't be useful for downstream analyses
  drop_na()
```

```
## Joining with `by = join_by(family)`
```

### Estimating net diversification rates using the method-of-moments estimator

With the species diversity, crown ages, and stem ages, we could
estimate net diversification rates using the method-of-moments
estimator. We examined three extinction fractions (\(\varepsilon\)): low (\(\varepsilon\) = 0), moderate (\(\varepsilon\) = 0.5), and high (\(\varepsilon\) = 0.9).

```
ext.rate<-c(0, 0.5, 0.9)
names(ext.rate)<-c('low', 'med', 'high')
###calculate diversification rates using crown ages and save to a dataframe
crown.rates<-data.frame(lapply(ext.rate, function(x) {
  (log(
    0.5 * fam.dat$n * (1 - x ^ 2) +
      2 * x + 0.5 * (1 - x) * sqrt(
        fam.dat$n * (
          fam.dat$n * x ^ 2 - 8 * x +
            2 * fam.dat$n *
            x +
            fam.dat$n
        )
      )
  ) - log(2)) / fam.dat$crown.age
}))
colnames(crown.rates)<-paste('crown', colnames(crown.rates), sep = '_')

###calculate diversification rates using stem ages and save to a dataframe
stem.rates<-data.frame(lapply(ext.rate, function(x) {
  log(fam.dat$n * (1 - x) + x) / fam.dat$stem.age
}))
colnames(stem.rates)<-paste('stem', colnames(stem.rates), sep = '_')

###concatenate crown/stem ages and crown/stem diversification rates into a single
###dataframe
fam.dat<-cbind(fam.dat, crown.rates, stem.rates)
```

In addition to net diversification rates derived from the
method-of-moments estimator, we also considered net diversification
rates derived from the birth-death estimator. Rather than calculating
them here, we utilized previously published data in Moen et al. (2021).
Note that Moen et al. (2021) were unable to estimate rates for several
families (Ascaphidae, Pelodtyidae, Heleophrynidae, Sooglossidae,
Conrauidae) because these families have too few species (or too few
sampled in the phylogeny) to estimate reasonable rates. For these
families, we assumed that if such rates were estimable, they would be
similar to the method-of-moments estimates (see our Supplementary Table
2, which shows high correlation between results from the two methods).
Thus, we substituted the rates derived from the latter estimators using
stem ages and a moderate extinction fraction.

```
fam.dat<-left_join(fam.dat, bd.rates %>% select(family, bd_rates))
```

```
## Joining with `by = join_by(family)`
```

```
fam.dat[!complete.cases(fam.dat), 'bd_rates']<-fam.dat[!complete.cases(fam.dat), 'stem_med']
```

For downstream analyses, we mean-centered and scaled all rates
(morphological and diversification) to unit variance.

```
#center and scale
fam.dat.cs<-fam.dat %>%
  relocate(family, .before = stem.age) %>%
  mutate(ratio.sigma = log(ratio.sigma),
         across(crown_low:crown_high, ~log(.x +1)),
         across(stem_low:bd_rates, ~ log(.x))) %>%
  mutate(across(ratio.sigma:bd_rates, ~ scale(.x, center = T) %>% as.vector))
```

## Characterizing anuran morphospace and quantifying morphological diversity

We characterized the morphological diversity of anurans using a
phylogenetically informed PCA, as implemented in
`phytools::phyl.pca`. The function requires an ultrametric
tree and a named matrix (or dataframe) of traits. Here, we modeled the
traits as evolving under Brownian motion. Because we standardized them
to be on a single scale—linear and size-corrected—we calculated the
principal components using a covariance matrix. To show that neither
method of size-correction (i.e., log ratios *vs.* residuals) nor
analysis (i.e., phylogenetically informed *vs.* not) influenced
our interpretation of the results, we repeated a phylogenetically
informed PCA on the residuals of SVL (`mean.resid.dat`) and
performed a standard (i.e., phylogenetically naive) PCA on ratio data
(`mean.ratio.dat`).

```
ppca<-phyl.pca(tree = sp.tree, 
               Y = mean.ratio.dat %>% column_to_rownames('species') %>% select(head.L:crus.v),
               method = 'BM', mode = 'cov')

ppca.resid<-phyl.pca(tree = sp.tree,
                     Y = mean.resid.dat,
                     method = 'BM', mode = 'cov')

spca<-princomp(x = mean.ratio.dat %>% column_to_rownames('species') %>% select(head.L:crus.v))
```

To assess similarity among methods, we performed a Mantel test
between the eigenvector matrices from each analysis. However, we note
that these eigenvectors may have arbitrarily opposite signs (i.e., their
vector elements positive or negative) between each analysis, which would
affect the strength of the correlation. Thus a quick inspection of the
eigenvectors was necessary.

```
ppca.evec<-ppca$Evec
ppca.resid.evec<-ppca.resid$Evec
spca.evec<-spca$loadings[1:10,] #the eigenvectors here are referred to as loadings.
round(ppca.evec, 3)
```

```
##                 PC1    PC2    PC3    PC4    PC5    PC6    PC7    PC8    PC9
## head.L        0.031 -0.029 -0.019 -0.246  0.676  0.267  0.165 -0.056  0.609
## head.W       -0.006  0.011  0.091 -0.218  0.439  0.187  0.369  0.215 -0.709
## front.limb.L  0.054 -0.045 -0.014 -0.152  0.313 -0.277 -0.378 -0.416 -0.314
## hind.limb.L   0.075 -0.003  0.048 -0.356  0.092 -0.155 -0.391 -0.398 -0.012
## foot.web      0.821  0.551 -0.095  0.094  0.001  0.031  0.053 -0.027 -0.014
## foot.pad      0.361 -0.547 -0.150 -0.091 -0.149  0.616 -0.342  0.094 -0.097
## hand.pad      0.366 -0.596 -0.223  0.071  0.074 -0.556  0.361  0.021  0.052
## inner.mtt     0.190 -0.185  0.878  0.363  0.132 -0.010 -0.080 -0.014  0.038
## thigh.v       0.099  0.054  0.194 -0.522 -0.051 -0.307 -0.281  0.691  0.109
## crus.v        0.083 -0.041  0.314 -0.562 -0.443  0.098  0.453 -0.362  0.043
##                PC10
## head.L       -0.084
## head.W        0.178
## front.limb.L -0.619
## hind.limb.L   0.722
## foot.web     -0.010
## foot.pad     -0.059
## hand.pad      0.088
## inner.mtt     0.031
## thigh.v      -0.108
## crus.v       -0.183
```

```
round(ppca.resid.evec, 3)
```

```
##                 PC1    PC2    PC3    PC4    PC5    PC6    PC7    PC8    PC9
## head.L        0.034 -0.030 -0.020 -0.253  0.675 -0.258 -0.195 -0.035  0.608
## head.W       -0.018  0.011  0.068 -0.212  0.452 -0.183 -0.232 -0.303 -0.747
## front.limb.L  0.055 -0.046 -0.013 -0.158  0.305  0.265  0.256  0.575 -0.229
## hind.limb.L   0.081 -0.004  0.059 -0.378  0.066  0.139  0.168  0.482 -0.033
## foot.web      0.827  0.544 -0.068  0.093  0.001 -0.033 -0.064  0.004 -0.019
## foot.pad      0.362 -0.550 -0.136 -0.090 -0.152 -0.617  0.358  0.022 -0.070
## hand.pad      0.366 -0.599 -0.214  0.082  0.078  0.559 -0.334 -0.137  0.018
## inner.mtt     0.166 -0.184  0.907  0.311  0.118  0.011  0.049  0.027  0.033
## thigh.v       0.092  0.056  0.172 -0.537 -0.063  0.322  0.492 -0.547  0.105
## crus.v        0.058 -0.040  0.265 -0.561 -0.443 -0.110 -0.571  0.159  0.024
##                PC10
## head.L       -0.048
## head.W        0.097
## front.limb.L -0.598
## hind.limb.L   0.749
## foot.web     -0.018
## foot.pad     -0.047
## hand.pad      0.069
## inner.mtt     0.027
## thigh.v      -0.103
## crus.v       -0.225
```

```
round(spca.evec, 3)
```

```
##              Comp.1 Comp.2 Comp.3 Comp.4 Comp.5 Comp.6 Comp.7 Comp.8 Comp.9
## head.L        0.026  0.062  0.204  0.478  0.565  0.110  0.027  0.190  0.424
## head.W        0.001 -0.003  0.268  0.224  0.457  0.353 -0.065 -0.305 -0.512
## front.limb.L -0.004  0.077  0.060  0.073  0.170 -0.660  0.223 -0.106 -0.545
## hind.limb.L   0.060  0.052  0.108  0.395 -0.061 -0.455  0.154  0.427  0.116
## foot.web      0.979 -0.161 -0.103 -0.031  0.044  0.011  0.006 -0.012 -0.027
## foot.pad      0.115  0.624  0.073  0.107 -0.281  0.339  0.611  0.023 -0.089
## hand.pad      0.128  0.738  0.046 -0.121  0.082 -0.211 -0.576 -0.146  0.114
## inner.mtt     0.057 -0.060  0.786 -0.557  0.081 -0.066  0.144  0.133  0.122
## thigh.v       0.038 -0.137  0.314  0.340 -0.346 -0.169  0.044 -0.719  0.313
## crus.v        0.050 -0.075  0.370  0.329 -0.474  0.170 -0.440  0.347 -0.336
##              Comp.10
## head.L         0.422
## head.W        -0.430
## front.limb.L   0.404
## hind.limb.L   -0.629
## foot.web       0.019
## foot.pad       0.073
## hand.pad      -0.070
## inner.mtt     -0.027
## thigh.v       -0.004
## crus.v         0.257
```

An inspection of the eigenvectors shows that for
`ppca.resid`, PC6, 7, and 8 had roughly opposite signs when
compared to the corresponding eigenvectors from `ppca`.
Similarly, components 2, 4, 7, 8, and 10 of `spca` were
roughly opposite to the corresponding eigenvectors from
`ppca`. We thus multiplied each of these vectors by -1.
Because each PC axis is independent of other axes, its signs were
arbitrary relative to other columns.

```
ppca.resid.evec[, 6:8]<-ppca.resid.evec[, 6:8] * -1
spca.evec[, c(2, 4, 7, 8, 10)]<-spca.evec[, c(2, 4, 7, 8, 10)] * -1
```

Now that the eigenvectors were roughly similar, we used a Mantel test
(as implemented in `vegan::mantel`) to assess the strength of
their correlations. We can see from these tests that the eigenvectors
derived from all methods were strongly correlated, and thus yielded
similar interpretations.

```
mantel(ppca.evec, ppca.resid.evec, permutation = 10000)
```

```
## 
## Mantel statistic based on Pearson's product-moment correlation 
## 
## Call:
## mantel(xdis = ppca.evec, ydis = ppca.resid.evec, permutations = 10000) 
## 
## Mantel statistic r: 0.9875 
##       Significance: 9.999e-05 
## 
## Upper quantiles of permutations (null model):
##   90%   95% 97.5%   99% 
## 0.197 0.253 0.302 0.366 
## Permutation: free
## Number of permutations: 10000
```

```
mantel(ppca.evec, spca.evec, permutation = 10000)
```

```
## 
## Mantel statistic based on Pearson's product-moment correlation 
## 
## Call:
## mantel(xdis = ppca.evec, ydis = spca.evec, permutations = 10000) 
## 
## Mantel statistic r: 0.8856 
##       Significance: 9.999e-05 
## 
## Upper quantiles of permutations (null model):
##   90%   95% 97.5%   99% 
## 0.192 0.251 0.301 0.365 
## Permutation: free
## Number of permutations: 10000
```

### Quantifying *n-*dimensional morphological diversity

Using the species scores from `ppca`, we could map each
species’ placement in up to a ten-dimensional morphospace (i.e., one
dimension for each PC-axis). This space represents the total
morphological diversity of Anura. We used two methods to define the
limits of this space and to quantify its volume in
*n-*dimensions: 1. convex hulls and 2. hypervolumes.

Convex hulls are effectively *n-*dimensional ranges, as their
limits are defined by the outer-most points for all dimensions. This
means that if there are any significant gaps between points (or species
in our case), convex hulls will over-estimate the total volume occupied
by a collection of points.

Hypervolume methods attempt to alleviate over-estimation of volume by
employing machine-learning algorithms to determine the presence of not
only outliers, but also holes. Holes in this case are gaps between
observations in *n-*dimensional space that do not necessarily
occur on the fringes of the observations.

The outputs of these methods are intuitive. However, their
convenience comes at the cost of computational burden. Hypervolume
methods in particular will take longer as dimensionality and the number
of observations increase. Here, we aimed to optimize the amount of
variation captured while balancing computational time by including a
subset of the PC-axes that we computed in `ppca`. We
determined which axes to use by visually inspecting a scree plot.

```
data.frame(axis = factor(1:10),
           eval = diag(ppca$Eval),
           prop.var = diag(ppca$Eval)/sum(diag(ppca$Eval))) %>%
  mutate(cum.sum = cumsum(prop.var)) %>%
  ggplot(aes(x = axis, y = prop.var, group = 1, label = round(cum.sum, 3))) +
  geom_point(pch = 16, size = 2) +
  geom_text(position = position_nudge(x = 0.05, y = 0.025)) +
  geom_line() +
  xlab('PC-Axis') +
  ylab('Proportion of Variance Explained') +
  theme_bw() +
  theme(aspect.ratio = 1)
```

The y-position of the dot in the above plot shows the amount of
variance explained by each PC-axis. The values above each point show the
cumulative sum of the proportion of the variance explained up to that
PC-axis. The line connecting each point gives us a sense of the
steepness of the drop in variance explained by each PC-axis. For
downstream analysis, we considered the first 5 PC axes, which accounted
for approximately 92% of the total variance in our morphological data
set.

```
ppca.scores<-data.frame(ppca$S) %>%
  rownames_to_column('species') %>%
  left_join(mean.ratio.dat %>% select(species, family, microhabitat))
```

```
## Joining with `by = join_by(species)`
```

```
ppca.scores[which(ppca.scores$microhabitat == ''), 'microhabitat']<-NA
ppca.scores$microhabitat<-factor(ppca.scores$microhabitat,
                                 levels = c('torrential ', 'aquatic', 'semi.aquatic',
                                            'arboreal', 'semi.arboreal',
                                            'burrowing', 'semi.burrowing', 'terrestrial'))
```

We then visualized the morphospace defined by the first two pPC axes,
as we did in Figure 1 of the manuscript. We also visualized these data
related to anuran ecomorphs, as we did in Supplementary Figure 1. Note
that `ppca.scores` contains all taxa from our morphological
dataset—this means that there are some taxa that are the sole
representatives of their families. We removed these taxa for
morphological diversity assessment, as explained above.

```
#create sub-plots for figure 1
hylid.plot<-ggplot(ppca.scores, aes(x = PC1, y = PC2)) +
  geom_hex(bins = 15, aes(fill = after_stat(count)), color = 'grey78') +
  scale_fill_gradientn(colors =c('white', 'black')) +
  labs(fill = guide_colorbar(title = 'Count', title.position = 'top')) +
  new_scale_fill() +
  geom_point(data = ppca.scores %>% filter(family == 'Hylidae'),
             aes(x = PC1, y = PC2), fill = 'dodgerblue', shape = 21, size = 2) +
  labs(x = 'pPC-1 (41.19%)',
       y = 'pPC-2 (28.19%)') +
  ggtitle(label = 'a. Hylidae (n = 207 of 1036)') +
  theme_bw() +
  theme(aspect.ratio = 1,
        axis.text.x = element_blank(),
        axis.title.x = element_blank())

ranid.plot<-ggplot(ppca.scores, aes(x = PC1, y = PC2)) +
  geom_hex(bins =15, aes(fill = after_stat(count)), color = 'grey78') +
  scale_fill_gradientn(colors =c('white', 'black')) +
  labs(fill = guide_colorbar(title = 'Count', title.position = 'top')) +
  new_scale_fill() +
  geom_point(data = ppca.scores %>% filter(family == 'Ranidae'),
             aes(x = PC1, y = PC2), fill = 'dodgerblue', shape = 21, size = 2) +
  labs(x = 'pPC-1 (41.19%)',
       y = 'pPC-2 (28.19%)') +
  ggtitle(label = 'b. Ranidae (n = 103 of 434)') +
  theme_bw() +
  theme(aspect.ratio = 1,
        axis.text = element_blank(),
        axis.title = element_blank())

pyxi.plot<-ggplot(ppca.scores, aes(x = PC1, y = PC2)) +
  geom_hex(bins =15, aes(fill = after_stat(count)), color = 'grey78') +
  scale_fill_gradientn(colors =c('white', 'black')) +
  labs(fill = guide_colorbar(title = 'Count', title.position = 'top')) +
  new_scale_fill() +
  geom_point(data = ppca.scores %>% filter(family == 'Pyxicephalidae'),
             aes(x = PC1, y = PC2), fill = 'dodgerblue', shape = 21, size = 2) +
  labs(x = 'pPC-1 (41.19%)',
       y = 'pPC-2 (28.19%)') +
  ggtitle(label = 'c. Pyxicephalidae (n = 23 of 87)') +
  theme_bw() +
  theme(aspect.ratio = 1)


bomb.plot<-ggplot(ppca.scores, aes(x = PC1, y = PC2)) +
  geom_hex(bins =15, aes(fill = after_stat(count)), color = 'grey78') +
  scale_fill_gradientn(colors =c('white', 'black')) +
  labs(fill = guide_colorbar(title = 'Count', title.position = 'top')) +
  new_scale_fill() +
  geom_point(data = ppca.scores %>% filter(family == 'Bombinatoridae'),
             aes(x = PC1, y = PC2), fill = 'dodgerblue', shape = 21, size = 2) +
  labs(x = 'pPC-1 (41.19%)',
       y = 'pPC-2 (28.19%)') +
  ggtitle(label = 'd. Bombinatoridae (n = 6 of 10)') +
  theme_bw() +
  theme(aspect.ratio = 1,
        axis.text.y = element_blank(),
        axis.title.y = element_blank())

(fam.morpho.plot<-hylid.plot +
  ranid.plot +
  pyxi.plot +
  bomb.plot + 
  plot_layout(nrow = 2, guides = 'collect'))
```

```
#create sub-plots for supplementary figure 1
burr.pc34<-ggplot() +
  ggtitle('c') +
  geom_point(
    data = ppca.scores %>% filter(!grepl('-', microhabitat)),
    aes(x = PC3, y = PC4, fill = microhabitat),
    alpha = 0.3,
    shape = 21,
    size = 2
  ) +
  geom_point(
    data = ppca.scores %>% filter(!grepl('-', microhabitat),
                                  grepl('burrow', microhabitat)),
    aes(x = PC3, y = PC4, fill = microhabitat),
    shape = 21,
    size = 2
  ) +
  scale_fill_viridis(
    discrete = T,
    na.value = 'white',
    option = 'turbo',
    labels = c(
      'Torrential',
      'Aquatic',
      'Semi-aquatic',
      'Arboreal',
      'Semi-arboreal',
      'Burrowing',
      'Semi-burrowing',
      'Terrestrial',
      'Unknown'
    )
  ) +
  labs (fill = 'Microhabitat',
        x = 'pPC-3 (11.47%)',
        y = 'pPC-4 (7.24%)') +
  guides(fill = guide_legend(title.position = 'top', order = 0)) +
  theme_bw() +
  theme(
    aspect.ratio = 1,
    legend.position = 'none',
    plot.margin = unit(c(0, 0.1, 0, 0), 'cm')
  )

aqua.pc12<-ggplot() +
  theme_bw() +
  ggtitle('a') +
  geom_point(
    data = ppca.scores %>% filter(!grepl('-', microhabitat)),
    aes(x = PC1, y = PC2, fill = microhabitat),
    alpha = 0.3,
    shape = 21,
    size = 2
  ) +
  geom_point(
    data = ppca.scores %>% filter(!grepl('-', microhabitat),
                                  grepl('aquatic', microhabitat)),
    aes(x = PC1, y = PC2, fill = microhabitat),
    shape = 21,
    size = 2
  ) +
  geom_point(
    data = ppca.scores %>% filter(!grepl('-', microhabitat),
                                  grepl('torrential', microhabitat)),
    aes(x = PC1, y = PC2, fill = microhabitat),
    shape = 21,
    size = 2
  ) +
  scale_fill_viridis(
    discrete = T,
    na.value = 'white',
    option = 'turbo',
    labels = c(
      'Torrential',
      'Aquatic',
      'Semi-aquatic',
      'Arboreal',
      'Semi-arboreal',
      'Burrowing',
      'Semi-burrowing',
      'Terrestrial',
      'Unknown'
    )
  ) +
  labs (fill = 'Microhabitat',
        y = 'pPC-2 (28.19%)',
        x = 'pPC-1 (41.20%)') +
  guides(fill = guide_legend(title.position = 'top', order = 0)) +
  theme(
    aspect.ratio = 1,
    legend.position = 'none',
    plot.margin = unit(c(0, 0.1, 0, 0), 'cm')
  )

arbo.pc12<-ggplot() +
  theme_bw() +
  ggtitle('b') +
  geom_point(
    data = ppca.scores %>% filter(!grepl('-', microhabitat)),
    aes(x = PC1, y = PC2, fill = microhabitat),
    alpha = 0.3,
    shape = 21,
    size = 2
  ) +
  geom_point(
    data = ppca.scores %>% filter(!grepl('-', microhabitat),
                                  grepl('boreal', microhabitat)),
    aes(x = PC1, y = PC2, fill = microhabitat),
    shape = 21,
    size = 2
  ) +
  scale_fill_viridis(
    discrete = T,
    na.value = 'white',
    option = 'turbo',
    labels = c(
      'Torrential',
      'Aquatic',
      'Semi-aquatic',
      'Arboreal',
      'Semi-arboreal',
      'Burrowing',
      'Semi-burrowing',
      'Terrestrial',
      'Unknown'
    )
  ) +
  labs (fill = 'Microhabitat',
        y = 'pPC-2 (28.19%)',
        x = 'pPC-1 (41.20%)') +
  guides(fill = guide_legend(title.position = 'top', order = 0)) +
  theme(
    aspect.ratio = 1,
    legend.position = 'none',
    plot.margin = unit(c(0, 0.1, 0, 0), 'cm')
  )

terr.pc12<-ggplot() +
  theme_bw() +
  ggtitle('d') +
  geom_point(
    data = ppca.scores %>% filter(!grepl('-', microhabitat)),
    aes(x = PC1, y = PC2, fill = microhabitat),
    alpha = 0.3,
    shape = 21,
    size = 2
  ) +
  geom_point(
    data = ppca.scores %>% filter(!grepl('-', microhabitat),
                                  grepl('terr', microhabitat)),
    aes(x = PC1, y = PC2, fill = microhabitat),
    shape = 21,
    size = 2
  ) +
  scale_fill_viridis(
    discrete = T,
    na.value = 'white',
    option = 'turbo',
    labels = c(
      'Torrential',
      'Aquatic',
      'Semi-aquatic',
      'Arboreal',
      'Semi-arboreal',
      'Burrowing',
      'Semi-burrowing',
      'Terrestrial',
      'Unknown'
    )
  ) +
  labs (fill = 'Microhabitat',
        y = 'pPC-2 (28.19%)',
        x = 'pPC-1 (41.20%)') +
  guides(fill = guide_legend(title.position = 'top', order = 0)) +
  theme(
    aspect.ratio = 1,
    legend.position = 'right',
    plot.margin = unit(c(0, 0.1, 0, 0), 'cm')
  )
```

```
(micro.morph.plot<-aqua.pc12 + 
  arbo.pc12 + 
  burr.pc34 + 
  terr.pc12)
```

```
#remove taxa
taxa_remove<-ppca.scores %>%
  group_by(family) %>% 
  summarise(count = n()) %>% 
  filter(count < 2) %>%
  pull(family)

ppca.scores2<-ppca.scores %>%
  filter(!family %in% taxa_remove)
```

With the monotypic families excised, we first estimated a
five-dimensional convex hull for 1226 species. We used
`geometry::convhull`, which utilizes the qhull algorithm.

```
frog.ch<-convhulln(p = ppca.scores2[,2:6], 'FA')
```

The ‘`FA`’ option in the above code specifies that the
`convhull` output will contain area and volume
measurements.

Next, used `hypervolume::hypvervolume_svm` to estimate a
five-dimensional hypervolume using the same data as we did to estimate
the five-dimensional convex hull. The hypervolume method takes
substantially longer than estimating the convex hull. Furthermore, it is
a stochastic method and each run generates slightly different results.
For these reasons, we provide here the code we used to generate results
presented in the main manuscript, but include the resulting
`frog.hv` hypervolume object and all other hypervolumes that
we generated downstream. All these hypervolumes are included in a
separate file called ‘frog\_hypervolumes.RData’ and have object names
that end with `.hv`.

Because this hypervolume data file is large (>850 MB), you may not
want to load it. However, the tables we generated from these objects
were pre-loaded into this workspace with other data at the beginning,
so you can instead just type the table names directly to see their
results. These pre-loaded tables also allow you to generate figures
based on the hypervolumes.

```
#frog.hv<-hypervolume_svm(data = ppca.scores2[,2:6])
```

## Characterizing the radiation space

We next characterized the radiation space and quantified quadrant
morphological and species diversity. We did this with the
`get.vol` function. This is a custom wrapper function for
`geometry::convhulln` and
`hypervolume::hypvervolume_svm` we wrote to work specifically
with the dataframes created in this study. It takes ppca scores
(`scores`) and centered and scaled (morphological and net
diversification) rates (`rates`) as data inputs. It takes
additional arguments for how clades are defined (`clades`),
whether or not the median of the rates should be used to define the
radiation space (`med`), which data set is being used
(`dataset`), which diversification rate column
(`column`) to work with, and which method should be used to
estimate *n-*dimensional morphological diversity
(`vol`). This function estimates the *n-*dimensional
convex hull (`vol = 'conv'`) or hypervolume
(`vol = 'hyper'`) of each quadrant of the radiation space.
Each quadrant is bounded by either the means (`med = F`) or
the medians (`med = T`) of the morphological rate and net
diversification rate of the clade-level data. Each clade is assigned to
a quadrant based simply on whether it has rates greater or less than the
bounding values of the morphological and net diversification rate. All
species that belong to the clades within a quadrant are subset together,
and their ppca scores are used to estimate the *n-*dimensional
convex hull or hypervolume. We repeat this for each quadrant. In the
case that there are an odd number of clades sampled and the median is
used to estimate bounds, the species that belong to the median clade are
randomly and evenly split between quadrants that the clade straddles.
The function outputs the convex-hull or hypervolume object for each
quadrant and a table summarizing the volume and number of species
contained within each quadrant.

We used `get.vol` to estimate the five-dimensional convex
hull for quadrants of radiation spaces in which net diversfiication
rates were estimated under all extinction fractions for both crown and
stem ages, using the means as bounding values. We output the resulting
objects into a list and created a table of the results.

```
#estimate five-dimensional convex hulls for each quadrant
quads.ch<-list(
  get.vol(rates = fam.dat.cs, scores = ppca.scores2, clades = 'family', dataset = 'family', column = 'crown_low', vol = 'conv'),
  get.vol(rates = fam.dat.cs, scores = ppca.scores2, clades = 'family', dataset = 'family', column = 'crown_med', vol = 'conv'),
  get.vol(rates = fam.dat.cs, scores = ppca.scores2, clades = 'family', dataset = 'family', column = 'crown_high', vol = 'conv'),
  get.vol(rates = fam.dat.cs, scores = ppca.scores2, clades = 'family', dataset = 'family', column = 'stem_low', vol = 'conv'),
  get.vol(rates = fam.dat.cs, scores = ppca.scores2, clades = 'family', dataset = 'family', column = 'stem_med', vol = 'conv'),
  get.vol(rates = fam.dat.cs, scores = ppca.scores2, clades = 'family', dataset = 'family',column = 'stem_high', vol = 'conv')
)
quads.ch.table<-bind_rows(lapply(quads.ch, function(x) x$table))
quads.ch.table<-bind_rows(lapply(quads.ch, function(x) x$table))
quads.ch.table$vol.prop<-quads.ch.table$vol/frog.ch$vol
quads.ch.table$n.prop<-quads.ch.table$n/quads.ch.table %>% group_by(div.rate) %>% summarise(tot = sum(n)) %>% pull(tot) %>% unique
```

Below, we show the code we used to generate the hypervolumes of the
radiation space, but comment it out as running this code takes a while.
Instead, here we simply called on the `quads.hv` object and
extracted the resulting tables and bound the rows together.

```
# quads.hv<-list(
#   get.vol(rates = fam.dat.cs, scores = ppca.scores2, clades = 'family', dataset = 'family', column = 'crown_low', vol = 'hyper'),
#   get.vol(rates = fam.dat.cs, scores = ppca.scores2, clades = 'family', dataset = 'family', column = 'crown_med', vol = 'hyper'),
#   get.vol(rates = fam.dat.cs, scores = ppca.scores2, clades = 'family', dataset = 'family', column = 'crown_high', vol = 'hyper'),
#   get.vol(rates = fam.dat.cs, scores = ppca.scores2, clades = 'family', dataset = 'family', column = 'stem_low', vol = 'hyper'),
#   get.vol(rates = fam.dat.cs, scores = ppca.scores2, clades = 'family', dataset = 'family', column = 'stem_med', vol = 'hyper'),
#   get.vol(rates = fam.dat.cs, scores = ppca.scores2, clades = 'family', dataset = 'family',column = 'stem_high', vol = 'hyper')
# )
# quads.hv.table<-bind_rows(lapply(quads.hv, function(x) x$table))
# quads.hv.table$vol.prop<-quads.hv.table$vol/get_volume(frog.hv)
# quads.hv.table$n.prop<-quads.hv.table$n/quads.hv.table %>% group_by(div.rate) %>% summarise(tot = sum(n)) %>% pull(tot) %>% unique
```

Next, we estimated a five-dimensional convex hull and hypervolume for
each family. The minimum number of observations necessary to estimate
these objects is *n + 1*, where *n* is the number of
dimensions. For this reason, we further subset the ppca scores by
families for which we measured at least six species. We then extracted
the volume measurements from the convex hulls and hypervolumes and
arranged them in a table. We then took the 5th root of the volumes,
mean-centered, and scaled the variances to unity.

```
#create a vector of families with at least 6 species measured
fams<-ppca.scores2 %>% 
  group_by(family) %>% 
  summarise(n = n()) %>% 
  filter(n >= 6) %>%
  pull(family)

#estimate convex hulls and save output as a list
fam.ch<-lapply(fams, function(x) convhulln(p = ppca.scores2 %>%
                                             filter(family == x) %>%
                                             select(PC1:PC5),
                                           'FA')
)
names(fam.ch)<-fams

#this is the code used to estimate hypervolumes
# fam.hv<-lapply(fams, function(x) hypervolume_svm(data = ppca.scores2 %>%
#                                                    filter(family == x) %>%
#                                                    select(PC1:PC5)
# )
# )
#names(fams.hv)<-fams

# vol.evo<-bind_rows(mapply(function(x,y) {
#   data.frame(ch = x$vol,
#              hv = get_volume(y)
#              )
# }, x = fam.ch, y = fam.hv, SIMPLIFY = F), .id = 'family') %>%
#   left_join(., fam.dat.cs %>% select(family, ratio.sigma)) %>%
#   mutate(ch = scale(ch^(1/5), center = T) %>% as.vector(),
#          hv = scale(hv^(1/5), center = T) %>% as.vector()) %>%
#   column_to_rownames('family')
```

## Correlations between net diversification and morphological rates

We next estimated pGLS correlations between rates of multivariate
morphological evolution and the volumes of the convex hulls and
hypervolumes. These correlations showed that both methods of quantifying
morphological diversity were strongly correlated with one another, and
thus could be similarly interpreted. We also showed that both of these
methods are correlated with rate of evolution.

```
(ch.hv.evo.cor<-gls.corr(vol.evo, tree = treedata(fam.tree, vol.evo, sort = T, warnings = F)$phy))
```

```
## $covariances
##                      ch          hv ratio.sigma
## ch          0.007849826 0.007745243 0.002612107
## hv          0.007745243 0.008023284 0.002479781
## ratio.sigma 0.002612107 0.002479781 0.001963832
## 
## $correlations
##                    ch        hv ratio.sigma
## ch          1.0000000 0.9759531   0.6652866
## hv          0.9759531 1.0000000   0.6247195
## ratio.sigma 0.6652866 0.6247195   1.0000000
## 
## $unbiased.cors
##                    ch        hv ratio.sigma
## ch          1.0000000 0.9769612    0.673348
## hv          0.9769612 1.0000000    0.633000
## ratio.sigma 0.6733480 0.6330000    1.000000
```

```
cor.pvalue(ch.hv.evo.cor$unbiased.cors, nrow(vol.evo), type = 'r.stat')
```

```
## $type
## [1] "r.stat"
## 
## $test.stat
##                    ch           hv ratio.sigma
## ch                Inf 2.288858e+01    4.553798
## hv          22.888577 2.372657e+08    4.088346
## ratio.sigma  4.553798 4.088346e+00         Inf
## 
## $P.value
##                       ch          hv  ratio.sigma
## ch          0.0000000000 0.000000000 0.0001184613
## hv          0.0000000000 0.000000000 0.0003947530
## ratio.sigma 0.0001184613 0.000394753 0.0000000000
```

We then visualized these correlations as Figure 2 in the
manuscript.

```
(vol.evo.plot<-ggplot(vol.evo, aes(x = ratio.sigma, y = ch, size = hv, fill = hv)) +
          geom_point(shape = 21) +
          scale_x_continuous(breaks = pretty_breaks(n = 10)) +
          scale_y_continuous(breaks = pretty_breaks(n = 10)) +
          scale_size_continuous(name = 'Hypervolume',
                                breaks = seq(from = -2.25, to = 1.75),
                                limits = c(-1.75, 2.75),
                                guide = guide_legend(reverse = T)) +
          scale_fill_viridis(name = 'Hypervolume',
                             breaks = seq(from = -2.25, to = 1.75)) +
          labs(y = 'Convex Hull',
               x = expression(paste('Morphological Rate ', (sigma^{2})))) +
          theme_bw() +
          theme(aspect.ratio = 1)
)
```

Similar to above, we then assessed the correlations among species
richness, rate of morphological evolution, and net diversification rates
of each family. We found that the species richness was strongly
correlated with net diversification rates, whereas rates of
morphological evolution were only weakly correlated with net
diversification rates.

```
div.sp.n.cor<-gls.corr(fam.dat.cs %>% column_to_rownames('family') %>% select(n:bd_rates) %>% mutate(n = log(n)),
                       tree = treedata(fam.tree, fam.dat.cs %>% column_to_rownames('family'), sort = T, warnings = F)$phy)
div.sp.n.cor$unbiased.cors
```

```
##                     n ratio.sigma crown_low crown_med crown_high  stem_low
## n           1.0000000   0.3287704 0.6586682 0.6927913  0.8277731 0.8763767
## ratio.sigma 0.3287704   1.0000000 0.1294209 0.1485581  0.2248468 0.3570861
## crown_low   0.6586682   0.1294209 1.0000000 0.9986093  0.9524091 0.7638684
## crown_med   0.6927913   0.1485581 0.9986093 1.0000000  0.9670682 0.7795742
## crown_high  0.8277731   0.2248468 0.9524091 0.9670682  1.0000000 0.8176913
## stem_low    0.8763767   0.3570861 0.7638684 0.7795742  0.8176913 1.0000000
## stem_med    0.8907437   0.3552341 0.7535887 0.7705657  0.8148234 0.9988130
## stem_high   0.9235028   0.3541983 0.7342098 0.7548568  0.8166469 0.9892041
## bd_rates    0.7384537   0.2372352 0.7406433 0.7513525  0.7671758 0.7683670
##              stem_med stem_high  bd_rates
## n           0.8907437 0.9235028 0.7384537
## ratio.sigma 0.3552341 0.3541983 0.2372352
## crown_low   0.7535887 0.7342098 0.7406433
## crown_med   0.7705657 0.7548568 0.7513525
## crown_high  0.8148234 0.8166469 0.7671758
## stem_low    0.9988130 0.9892041 0.7683670
## stem_med    1.0000000 0.9948489 0.7714132
## stem_high   0.9948489 1.0000000 0.7805558
## bd_rates    0.7714132 0.7805558 1.0000000
```

```
diag(div.sp.n.cor$unbiased.cors) <- NA # To get reasonable results, since diagonal is a variable on itself (correlation == 1)
cor.pvalue(div.sp.n.cor$unbiased.cors, nrow(fam.dat.cs), type = 'z.stat')
```

```
## $type
## [1] "z.stat"
## 
## $test.stat
##                     n ratio.sigma  crown_low crown_med crown_high  stem_low
## n                  NA   2.1595135  4.9992925  5.396765   7.469430  8.600948
## ratio.sigma  2.159514          NA  0.8231461  0.946569   1.446775  2.362488
## crown_low    4.999292   0.8231461         NA 22.990973  11.745264  6.358947
## crown_med    5.396765   0.9465690 22.9909735        NA  12.933278  6.604633
## crown_high   7.469430   1.4467750 11.7452641 12.933278         NA  7.272041
## stem_low     8.600948   2.3624881  6.3589469  6.604633   7.272041        NA
## stem_med     9.015747   2.3490734  6.2057087  6.461917   7.217689 23.492206
## stem_high   10.197238   2.3415791  5.9311680  6.224312   7.252159 16.495466
## bd_rates     5.989796   1.5295455  6.0203621  6.173105   6.409476  6.427828
##              stem_med stem_high bd_rates
## n            9.015747 10.197238 5.989796
## ratio.sigma  2.349073  2.341579 1.529546
## crown_low    6.205709  5.931168 6.020362
## crown_med    6.461917  6.224312 6.173105
## crown_high   7.217689  7.252159 6.409476
## stem_low    23.492206 16.495466 6.427828
## stem_med           NA 18.844360 6.475133
## stem_high   18.844360        NA 6.620491
## bd_rates     6.475133  6.620491       NA
## 
## $P.value
##                        n ratio.sigma    crown_low    crown_med   crown_high
## n                     NA  0.03081035 5.754107e-07 6.785323e-08 8.060219e-14
## ratio.sigma 3.081035e-02          NA 4.104249e-01 3.438584e-01 1.479600e-01
## crown_low   5.754107e-07  0.41042494           NA 0.000000e+00 0.000000e+00
## crown_med   6.785323e-08  0.34385844 0.000000e+00           NA 0.000000e+00
## crown_high  8.060219e-14  0.14795995 0.000000e+00 0.000000e+00           NA
## stem_low    0.000000e+00  0.01815272 2.031417e-10 3.985035e-11 3.541611e-13
## stem_med    0.000000e+00  0.01882020 5.445098e-10 1.033851e-10 5.286882e-13
## stem_high   0.000000e+00  0.01920236 3.007872e-09 4.836742e-10 4.101164e-13
## bd_rates    2.101040e-09  0.12612926 1.740273e-09 6.696175e-10 1.460208e-10
##                 stem_low     stem_med    stem_high     bd_rates
## n           0.000000e+00 0.000000e+00 0.000000e+00 2.101040e-09
## ratio.sigma 1.815272e-02 1.882020e-02 1.920236e-02 1.261293e-01
## crown_low   2.031417e-10 5.445098e-10 3.007872e-09 1.740273e-09
## crown_med   3.985035e-11 1.033851e-10 4.836742e-10 6.696175e-10
## crown_high  3.541611e-13 5.286882e-13 4.101164e-13 1.460208e-10
## stem_low              NA 0.000000e+00 0.000000e+00 1.294400e-10
## stem_med    0.000000e+00           NA 0.000000e+00 9.472867e-11
## stem_high   0.000000e+00 0.000000e+00           NA 3.580070e-11
## bd_rates    1.294400e-10 9.472867e-11 3.580070e-11           NA
```

In Figure 3 of the main manuscript, we visualized change in net
diversification and morphological rates along the branches of the
phylogeny, using a cophylo-style plot. Here we simply estimated
ancestral states for visualization purposes and made no inference from
them. Note that the scale positions are reversed from the main
manuscript, for which we readjusted them manually.

```
fam.tree$tip.label<-as.character(fam.tree$tip.label)
fit.stem_med<-fastAnc(treedata(fam.tree, fam.dat.cs %>% column_to_rownames('family'), sort = T, warnings = F)$phy, fam.dat.cs %>% pull(stem_med, name=family), vars=T, CI=T)
fit.ratio.sigma<-fastAnc(treedata(fam.tree, fam.dat.cs %>% column_to_rownames('family'), sort = T, warnings = F)$phy, fam.dat.cs %>% pull(ratio.sigma, name=family), vars=T, CI=T)
stem.td<-data.frame(node = tidytree::nodeid(treedata(fam.tree, fam.dat.cs %>% column_to_rownames('family'), sort = T, warnings = F)$phy, fam.dat.cs %>% pull(family)), trait = fam.dat.cs$stem_med)
sigma.td<-data.frame(node = tidytree::nodeid(treedata(fam.tree, fam.dat.cs %>% column_to_rownames('family'), sort = T, warnings = F)$phy, fam.dat.cs %>% pull(family)), trait = fam.dat.cs$ratio.sigma)
stem.nd<-data.frame(node = names(fit.stem_med$ace), trait = fit.stem_med$ace)
sigma.nd<-data.frame(node = names(fit.ratio.sigma$ace), trait = fit.ratio.sigma$ace)
d.stem<-rbind(stem.td, stem.nd)
d.sigma<-rbind(sigma.td, sigma.nd)
d.stem$node<-as.numeric(d.stem$node)
d.sigma$node<-as.numeric(d.sigma$node)
stem.tree<-full_join(treedata(fam.tree, fam.dat.cs %>% column_to_rownames('family'), sort = T, warnings = F)$phy, d.stem, by='node')
sigma.tree<-full_join(treedata(fam.tree, fam.dat.cs %>% column_to_rownames('family'), sort = T, warnings = F)$phy, d.sigma, by='node')
stem.tree.plot <-
  ggtree(stem.tree,
         aes(color = trait),
         lwd = 0.5,
         ladderize = F) +
  scale_color_viridis(
    option = 'viridis',
    guide = guide_colorbar(
      title = 'Diversification Rate',
      # title.position = 'left',
      # label.position = 'top',
      frame.colour = 'black',
      frame.linewidth = 1 
    ),
    limits =c(-3.3, 1.2)
  ) +
  theme(legend.position = 'top')
sigma.tree.plot<-ggtree(sigma.tree, aes(color=trait), lwd=1, ladderize=F) +
  scale_color_viridis(option = 'inferno')


d1<-stem.tree.plot$data
d2<-sigma.tree.plot$data
d2$x<-max(d2$x) - d2$x + max(d1$x) +180
stem.tree.plot +
  new_scale_color() +
  geom_tree(data = d2, aes(color = trait), lwd = 0.5) +
  scale_color_viridis(
    option = 'inferno',
    guide = guide_colorbar(
      title = 'Morphological Rate',
      title.position = 'right',
      label.position = 'top',
      frame.linewidth = 1,
      frame.colour = 'black'
    ),
    limits = c(-3, 2.3)
  ) +
  geom_tiplab(
    color = 'black',
    hjust = 'center',
    offset = 90,
    size = 2.5
  ) +
  theme_tree2() +
  scale_x_continuous(
    breaks = seq(0, 220, by = 20),
    labels = rev(seq(0, 220, by = 20)),
    name = 'Time (MYA)'
  ) +
  theme(
    legend.position = 'top',
    plot.title = element_text(hjust = 1),
    aspect.ratio = 1,
    legend.key.width = unit(0.5, 'cm'),
    legend.key.height = unit(0.25, 'cm')
  )
```

## Visualizing the radiation space

We further visualized the weak correlation of net diversification and
morphological rates among families using a scatter plot, as in Figure 4
in the manuscript. We used dot color to indicate hypervolume size for
each family. Note that we could only calculate 5-dimensional hypervolume
for families with more than six observations, so families with fewer
observations were set as grey.

```
(evo.div.rate.plot<-fam.dat.cs %>% 
  left_join(vol.evo %>% select(-ratio.sigma) %>% rownames_to_column('family')) %>%
  ggplot(aes(x = stem_med, y = ratio.sigma, fill = hv)) +
  geom_hline(yintercept = 0) +
  geom_vline(xintercept = 0) +
  geom_point(shape = 21, size = 3) +
  scale_x_continuous(breaks = pretty_breaks(n = 10), limits = c(-3.5, 3.5)) +
  scale_y_continuous(breaks = pretty_breaks(n = 10),limits = c(-3.5, 3.5)) +
  labs(x = 'Net diversification rate', y = expression(paste('Morphological Rate  ', (sigma^{2}))), fill = NULL) +
  scale_fill_viridis(name = 'Hypervolume') + 
  theme_bw() +
  theme(aspect.ratio = 1)
)
```

```
## Joining with `by = join_by(family)`
```

We also created a labeled version of this figure (Supplementary
Figure 2), with the the region encompassed within the rectangle zoomed
in and shown in the panel to the right. Here, a single point in the
adaptive radiation quadrant (Bufonidae) remains unlabeled due to space
constraints (i.e., green fill, just outside of the inset bounding
box).

```
lab.plot<-fam.dat.cs %>% 
  left_join(vol.evo %>% select(-ratio.sigma) %>% rownames_to_column('family')) %>%
  ggplot(aes(x = stem_med, y = ratio.sigma, fill = hv, label = family)) +
  geom_hline(yintercept = 0) +
  geom_vline(xintercept = 0) +
  geom_rect(aes(xmin = -0.75, xmax = 1.5, ymin = -0.25, ymax = 0.8),
            fill = NA, color = 'black') +
  geom_point(shape = 21, size = 3) +
  scale_x_continuous(breaks = pretty_breaks(n = 10), limits = c(-3.5, 3.5)) +
  scale_y_continuous(breaks = pretty_breaks(n = 10),limits = c(-3.5, 3.5)) +
  geom_text_repel(size = 2) +
  labs(x = 'Net diversification rate', y = expression(paste('Morphological Rate  ', (sigma^{2}))), fill = NULL) +
  ggtitle('a.') +
  scale_fill_viridis(name = 'Hypervolume') + 
  theme_bw() +
  theme(aspect.ratio = 1,
        axis.text = element_text(size = 9),
        axis.title = element_text(size = 9),
        plot.title = element_text(size = 9),
        legend.position = 'bottom')
```

```
## Joining with `by = join_by(family)`
```

```
inset.lab1<-fam.dat.cs %>% 
  left_join(vol.evo %>% select(-ratio.sigma) %>% rownames_to_column('family')) %>%
  ggplot(aes(x = stem_med, y = ratio.sigma, fill = hv, label = family)) +
  geom_hline(yintercept = 0) +
  geom_vline(xintercept = 0) +
  geom_point(shape = 21, size = 3) +
  scale_x_continuous(breaks = c(-1, -0.5, 0, 0.5, 1, 1.5), limits = c(-0.75, 1.5)) +
  scale_y_continuous(breaks = c(-0.5, 0, 0.5, 1), limits = c(-0.25, 0.8)) +
  scale_fill_viridis(name = 'Hypervolume') + 
  geom_text_repel(size = 2) +
  labs(x = 'Net diversification rate', y = expression(paste('Morphological Rate  ', (sigma^{2}))), fill = NULL) +
  ggtitle('b.') +
  theme_bw() +
  theme(aspect.ratio = 0.75,
        axis.text = element_text(size = 9),
        axis.title = element_text(size = 9),
        plot.title = element_text(size = 9),
        legend.position ='bottom')
```

```
## Joining with `by = join_by(family)`
```

```
(lab.evo.div.plot<-lab.plot + inset.lab1 + plot_layout(ncol = 2, guides = 'collect') & 
    theme(legend.position = 'bottom', legend.title = element_text(size = 9)))
```

```
## Warning: Removed 17 rows containing missing values (`geom_point()`).
```

```
## Warning: Removed 17 rows containing missing values (`geom_text_repel()`).
```

```
## Warning: ggrepel: 28 unlabeled data points (too many overlaps). Consider
## increasing max.overlaps
```

We next showed the percentage of the overall frog morphological
diversity represented by each quadrant. We retrieved these values from
`quads.hv.table` and `quads.ch.table` that we
created (or imported) earlier from the `get.vol` function.
Here we show what we presented in the manuscript. However, each full
table contains species and volume proportions for each quadrant for all
methods of net diversification rates we used in our analyses. Simply
calling on the tables will print them to the screen.

```
quads.hv.table %>% filter(div.rate == 'stem_med')
```

```
##      quad      vols    n div.rate   vol.prop     n.prop
## ar     AR 2.2386518 5526 stem_med 0.75428048 0.75091724
## anr   ANR 0.4593750  149 stem_med 0.15477959 0.02024732
## nanr NANR 0.0766889  139 stem_med 0.02583919 0.01888844
## nar   NAR 0.4192334 1545 stem_med 0.14125447 0.20994700
```

```
#to see convex hull proportions
#quads.ch.table %>% filter(div.rate == 'stem_med')
```

We also explored the effect of using crown ages and different
extinction fractions to estimate net diversification rates and how that
affected the distribution of the morphological and species diversity in
each quadrant. We showed this in Supplementary Figure 3, using the code
below. Note that this code produces the figure without the annotations,
which we called after generating the plot.

```
(crown_stem_evo.div.plot<-fam.dat.cs %>%
  select(family, ratio.sigma:stem_high) %>%
  pivot_longer(cols = c(crown_low:stem_high), names_to = 'div', values_to = 'rate') %>%
  separate(col = div, into = c('div', 'ex.frac'), sep = '_') %>%
  mutate(ex.frac = factor(ex.frac, levels = c('low', 'med', 'high'))) %>% 
  ggplot(aes(x = rate, y = ratio.sigma)) +
  geom_vline(xintercept = 0) +
  geom_hline(yintercept = 0) +
  geom_point(shape = 21, fill = 'grey') +
  facet_grid(ex.frac ~ div) +
  xlab('Diversification rate') +
  ylab(expression(paste('Morphological rate ', (sigma^{2})))) +
  scale_x_continuous(breaks = pretty_breaks(n = 6), limits = c(-3.35, 3.35)) +
  scale_y_continuous(breaks = pretty_breaks(n = 6), limits = c(-3.35, 3.35)) +
  theme_bw() +
  theme(aspect.ratio = 1,
        strip.background = element_blank(),
        axis.text = element_text(color = 'black'))
)
```

```
quads.hv.table
```

```
##           quad       vols    n   div.rate   vol.prop     n.prop
## ar...1      AR 2.09881331 5023  crown_low 0.70716399 0.68256557
## anr...2    ANR 0.96643006  652  crown_low 0.32562426 0.08859899
## nanr...3  NANR 0.09741985  224  crown_low 0.03282417 0.03043892
## nar...4    NAR 0.39916575 1460  crown_low 0.13449297 0.19839652
## ar...5      AR 2.06100266 4997  crown_med 0.69442425 0.67903248
## anr...6    ANR 0.90670059  678  crown_med 0.30549930 0.09213208
## nanr...7  NANR 0.09739290  224  crown_med 0.03281509 0.03043892
## nar...8    NAR 0.39848973 1460  crown_med 0.13426520 0.19839652
## ar...9      AR 2.08556908 5150 crown_high 0.70270154 0.69982335
## anr...10   ANR 0.95181729  525 crown_high 0.32070071 0.07134121
## nanr...11 NANR 0.11392845  237 crown_high 0.03838650 0.03220546
## nar...12   NAR 0.39551134 1447 crown_high 0.13326167 0.19662998
## ar...13     AR 2.24809293 5526   stem_low 0.75746154 0.75091724
## anr...14   ANR 0.45779164  149   stem_low 0.15424610 0.02024732
## nanr...15 NANR 0.05748231  126   stem_low 0.01936781 0.01712189
## nar...16   NAR 0.42131006 1558   stem_low 0.14195417 0.21171355
## ar...17     AR 2.23865178 5526   stem_med 0.75428048 0.75091724
## anr...18   ANR 0.45937501  149   stem_med 0.15477959 0.02024732
## nanr...19 NANR 0.07668890  139   stem_med 0.02583919 0.01888844
## nar...20   NAR 0.41923339 1545   stem_med 0.14125447 0.20994700
## ar...21     AR 2.26186403 5526  stem_high 0.76210151 0.75091724
## anr...22   ANR 0.46074001  149  stem_high 0.15523951 0.02024732
## nanr...23 NANR 0.07636655  139  stem_high 0.02573058 0.01888844
## nar...24   NAR 0.41808166 1545  stem_high 0.14086641 0.20994700
```

```
quads.ch.table
```

```
##           quad       vols    n   div.rate   vol.prop     n.prop
## ar...1      AR 2.70900771 5023  crown_low 0.62878100 0.68256557
## anr...2    ANR 1.20755838  652  crown_low 0.28028335 0.08859899
## nanr...3  NANR 0.11396019  224  crown_low 0.02645101 0.03043892
## nar...4    NAR 0.57537492 1460  crown_low 0.13354883 0.19839652
## ar...5      AR 2.70900771 4997  crown_med 0.62878100 0.67903248
## anr...6    ANR 1.20826368  678  crown_med 0.28044706 0.09213208
## nanr...7  NANR 0.11396019  224  crown_med 0.02645101 0.03043892
## nar...8    NAR 0.57537492 1460  crown_med 0.13354883 0.19839652
## ar...9      AR 2.72049435 5150 crown_high 0.63144713 0.69982335
## anr...10   ANR 1.08336382  525 crown_high 0.25145686 0.07134121
## nanr...11 NANR 0.12835287  237 crown_high 0.02979166 0.03220546
## nar...12   NAR 0.57465310 1447 crown_high 0.13338129 0.19662998
## ar...13     AR 2.87056268 5526   stem_low 0.66627912 0.75091724
## anr...14   ANR 0.50990238  149   stem_low 0.11835216 0.02024732
## nanr...15 NANR 0.06248179  126   stem_low 0.01450249 0.01712189
## nar...16   NAR 0.58202314 1558   stem_low 0.13509193 0.21171355
## ar...17     AR 2.87056268 5526   stem_med 0.66627912 0.75091724
## anr...18   ANR 0.50990238  149   stem_med 0.11835216 0.02024732
## nanr...19 NANR 0.10273513  139   stem_med 0.02384559 0.01888844
## nar...20   NAR 0.58130133 1545   stem_med 0.13492440 0.20994700
## ar...21     AR 2.87056268 5526  stem_high 0.66627912 0.75091724
## anr...22   ANR 0.50990238  149  stem_high 0.11835216 0.02024732
## nanr...23 NANR 0.10273513  139  stem_high 0.02384559 0.01888844
## nar...24   NAR 0.58130133 1545  stem_high 0.13492440 0.20994700
```

In Supplementary Figure 2, we also showed the effect of using median
values of net diversification and morphological rates to delimit
quadrant boundaries, as well as the effect of using net diversification
rates derived from the birth-death estimator. As above, the proportions
can be called from the tables created from `get.vol`.

```
quads.med.ch<-list(
  get.vol(rates = fam.dat.cs, scores = ppca.scores2, clades = 'family', dataset = 'family',  column = 'crown_low', vol = 'conv', med = T),
  get.vol(rates = fam.dat.cs, scores = ppca.scores2, clades = 'family', dataset = 'family', column = 'crown_med', vol = 'conv', med = T),
  get.vol(rates = fam.dat.cs, scores = ppca.scores2, clades = 'family', dataset = 'family', column = 'crown_high', vol = 'conv', med = T),
  get.vol(rates = fam.dat.cs, scores = ppca.scores2, clades = 'family', dataset = 'family', column = 'stem_low', vol = 'conv', med = T),
  get.vol(rates = fam.dat.cs, scores = ppca.scores2, clades = 'family', dataset = 'family', column = 'stem_med', vol = 'conv', med = T),
  get.vol(rates = fam.dat.cs, scores = ppca.scores2, clades = 'family', dataset = 'family', column = 'stem_high', vol = 'conv', med = T),
  get.vol(rates = fam.dat.cs, scores = ppca.scores2, clades = 'family', dataset = 'family', column = 'bd_rates', vol = 'conv', med = T)
)
quads.med.ch.table<-bind_rows(lapply(quads.med.ch, function(x) x$table))
quads.med.ch.table$vol.prop<-quads.med.ch.table$vol/frog.ch$vol
quads.med.ch.table$n.prop<-quads.med.ch.table$n/quads.med.ch.table %>% group_by(div.rate) %>% summarise(tot = sum(n)) %>% pull(tot) %>% unique

bd.ch<-get.vol(rates = fam.dat.cs, scores = ppca.scores2, clades = 'family', dataset = 'family',column = 'bd_rates', vol = 'conv')
bd.ch.table<-bd.ch$table
bd.ch.table$vol.prop<-bd.ch.table$vol/frog.ch$vol
bd.ch.table$n.prop<-bd.ch.table$n/bd.ch.table %>% group_by(div.rate) %>% summarise(tot = sum(n)) %>% pull(tot) %>% unique

# system.time(quads.med.hv<-list(
#   get.vol(rates = fam.dat.cs, scores = ppca.scores2, clades = 'family', dataset = 'family',  column = 'crown_low', vol = 'hyper', med = T),
#   get.vol(rates = fam.dat.cs, scores = ppca.scores2, clades = 'family', dataset = 'family', column = 'crown_med', vol = 'hyper', med = T),
#   get.vol(rates = fam.dat.cs, scores = ppca.scores2, clades = 'family', dataset = 'family', column = 'crown_high', vol = 'hyper', med = T),
#   get.vol(rates = fam.dat.cs, scores = ppca.scores2, clades = 'family', dataset = 'family', column = 'stem_low', vol = 'hyper', med = T),
#   get.vol(rates = fam.dat.cs, scores = ppca.scores2, clades = 'family', dataset = 'family', column = 'stem_med', vol = 'hyper', med = T),
#   get.vol(rates = fam.dat.cs, scores = ppca.scores2, clades = 'family', dataset = 'family', column = 'stem_high', vol = 'hyper', med = T),
#   get.vol(rates = fam.dat.cs, scores = ppca.scores2, clades = 'family', dataset = 'family', column = 'bd_rates', vol = 'hyper', med = T)
# )) # took 1459 seconds
# quads.med.hv.table<-bind_rows(lapply(quads.med.hv, function(x) x$table))
# quads.med.hv.table$vol.prop<-quads.med.hv.table$vol/get_volume(frog.hv)
# quads.med.hv.table$n.prop<-quads.med.hv.table$n/quads.med.hv.table %>% group_by(div.rate) %>% summarise(tot = sum(n)) %>% pull(tot) %>% unique

#bd.hv<-get.vol(rates = fam.dat.cs, scores = ppca.scores2, clades = 'family', dataset = 'family',column = 'bd_rates', vol = 'hyper')
# bd.hv.table<-bd.hv$table
# bd.hv.table$vol.prop<-bd.hv.table$vol/get_volume(frog.hv)
# bd.hv.table$n.prop<-bd.hv.table$n/bd.hv.table %>% group_by(div.rate) %>% summarise(tot = sum(n)) %>% pull(tot) %>% unique
  
  
(median_bd.rates.plot<-fam.dat.cs %>%
  left_join(vol.evo %>% select(-ratio.sigma) %>% rownames_to_column('family')) %>%
  ggplot(aes(x = stem_med, y = ratio.sigma, fill = hv)) +
  geom_hline(yintercept = median(fam.dat.cs$ratio.sigma)) +
  geom_vline(xintercept = median(fam.dat.cs$stem_med)) +
  geom_point(shape = 21, size = 3) +
  scale_x_continuous(breaks = pretty_breaks(10), limits = c(-3.5, 3.5)) +
  scale_y_continuous(breaks = pretty_breaks(10), limits = c(-3.5, 3.5)) +
  scale_fill_viridis() +
  labs(x = 'Net Diversification Rate (Stem age, moderate extinction rate)',
       y = expression(paste('Morphological Rate ', (sigma^{2}))),
       title = 'c. Medians as quadrant boundaries') +
  theme_bw() +
   theme(aspect.ratio = 1,
        axis.text = element_text(size = 9),
        axis.title = element_text(size = 9),
        legend.text = element_text(size = 9),
        legend.title = element_text(size = 9),
        plot.title = element_text(size = 9)) + 
fam.dat.cs %>%
  left_join(vol.evo %>% select(-ratio.sigma) %>% rownames_to_column('family')) %>%
  ggplot(aes(x = bd_rates, y = ratio.sigma, fill = hv)) +
  geom_hline(yintercept = 0) +
  geom_vline(xintercept = 0) +
  geom_point(shape = 21, size = 3) +
  scale_x_continuous(breaks = pretty_breaks(6), limits = c(-3, 3)) +
  scale_y_continuous(breaks = pretty_breaks(6), limits = c(-3, 3)) +
  scale_fill_viridis() +
  labs(x = 'Net Diversification Rate (Birth-death estimator)',
       y = expression(paste('Morphological Rate ', (sigma^{2}))),
       title = 'd. Means as quadrant boundaries') +
  theme_bw() +
  theme(aspect.ratio = 1,
        axis.text = element_text(size = 9),
        axis.title = element_text(size = 9),
        legend.text = element_text(size = 9),
        legend.title = element_text(size = 9),
        plot.title = element_text(size = 9)) +
  plot_layout(ncol = 2, guides = 'collect'))
```

```
## Joining with `by = join_by(family)`
## Joining with `by = join_by(family)`
```

```
quads.med.hv.table %>% filter(div.rate == 'stem_med')
```

```
##      quad      vols       n div.rate  vol.prop    n.prop
## ar     AR 1.8000955 4665.25 stem_med 0.6065155 0.6339516
## anr   ANR 1.0955614  424.25 stem_med 0.3691332 0.0576505
## nanr NANR 0.3305861  482.25 stem_med 0.1113861 0.0655320
## nar   NAR 0.5719343 1787.25 stem_med 0.1927048 0.2428659
```

```
quads.med.ch.table %>% filter(div.rate == 'stem_med')
```

```
##      quad      vols       n div.rate  vol.prop    n.prop
## ar     AR 2.6229603 4665.25 stem_med 0.6088088 0.6339516
## anr   ANR 0.9834305  424.25 stem_med 0.2282616 0.0576505
## nanr NANR 0.4614207  482.25 stem_med 0.1070992 0.0655320
## nar   NAR 0.6785829 1787.25 stem_med 0.1575042 0.2428659
```

```
bd.hv.table
```

```
##      quad       vols    n div.rate   vol.prop     n.prop
## ar     AR 2.39197891 5538 bd_rates 0.80594178 0.75254790
## anr   ANR 0.24034201  137 bd_rates 0.08097967 0.01861666
## nanr NANR 0.07630035  139 bd_rates 0.02570827 0.01888844
## nar   NAR 0.41713551 1545 bd_rates 0.14054762 0.20994700
```

```
bd.ch.table
```

```
##      quad      vols    n div.rate   vol.prop     n.prop
## ar     AR 3.0980241 5538 bd_rates 0.71907461 0.75254790
## anr   ANR 0.2507552  137 bd_rates 0.05820217 0.01861666
## nanr NANR 0.1027351  139 bd_rates 0.02384559 0.01888844
## nar   NAR 0.5813013 1545 bd_rates 0.13492440 0.20994700
```

## Using 80-, 100-, and 120-million-year-old time slices instead of named clades

We also investigated whether our results were sensitive to the
arbitrariness of named clades. We did this by creating time slices at
80-, 100-, and 120-million-year intervals. Our approach here was to
estimate species diversity (and thus net diversification rates) from the
full taxonomic tree of Jetz & Pyron (2018); by including all anuran
species, this full tree would give our time-sliced clades all species
described at the time the was estimated. In contrast, we estimated
morphological rates from `jp.tree`, which was the tree with
genetic data from Jetz & Pyron (2018) pruned to match our
morphological sampling.

We started first by creating two sets of these time-sliced trees, one
set from the taxonomic tree and one set from `jp.tree`. The
challenge here was that the number of clades extracted from each tree
was not equal—some clades occurred in the taxonomic tree but not on
`jp.tree`, as they lacked genetic data. Thus, we needed to
match clades between trees based on species membership while also taking
note of the species richness of the clade from the taxonomic tree.

```
#subset the tree to include only anurans (this tree contains all amphibians)
jp7k<-extract.clade(jp7k, node = findMRCA(jp7k, tips = c('Ascaphus_truei', 'Aglyptodactylus_madagascariensis')))
is.ultrametric(jp7k)
```

```
## [1] TRUE
```

```
#similar to the genetic tree, the taxonomic tree is not ultrametric due to numerical precision
jp7k<-force.ultrametric(jp7k)
```

```
## ***************************************************************
## *                          Note:                              *
## *    force.ultrametric does not include a formal method to    *
## *    ultrametricize a tree & should only be used to coerce    *
## *   a phylogeny that fails is.ultramtric due to rounding --   *
## *    not as a substitute for formal rate-smoothing methods.   *
## ***************************************************************
```

```
#again, similar to the genetic tree, rename several taxa
jp7k$tip.label[c(5248, 5250:5257)]<-gsub('Indirana_', 'Sallywalkerana_', jp7k$tip.label[c(5248, 5250:5257)])
jp7k$tip.label[5217]<-'Alcalus_baluensis'

#create a vector of species names from the morphological dataset
sp.in.dat<-mean.ratio.dat %>%
    pull(species)


#create time slices at 80, 100, and 120 million year intervals for both the taxonomic tree and from the original tree from the start of this markdown document
jp7k.80<-treeSlice(jp7k, slice = max(nodeHeights(jp7k))-80, trivial = F)
jp7k.100<-treeSlice(jp7k, slice = max(nodeHeights(jp7k))-100, trivial = F)
jp7k.120<-treeSlice(jp7k, slice = max(nodeHeights(jp7k))-120, trivial = F)
jp.tree.80<-treeSlice(jp.tree, slice = max(nodeHeights(jp.tree))-80, trivial = F)
jp.tree.100<-treeSlice(jp.tree, slice = max(nodeHeights(jp.tree))-100, trivial = F)
jp.tree.120<-treeSlice(jp.tree, slice = max(nodeHeights(jp.tree))-120, trivial = F)

#create a dataframe for each time slice of species names with which numbered clade they belong to
jp7k.taxa.80<-bind_rows(lapply(jp7k.80, function(x) data.frame(species = x$tip.label)), .id = 'clade') %>%
  mutate(clade = as.numeric(clade))
jp7k.taxa.100<-bind_rows(lapply(jp7k.100, function(x) data.frame(species = x$tip.label)), .id = 'clade') %>%
  mutate(clade = as.numeric(clade))
jp7k.taxa.120<-bind_rows(lapply(jp7k.120, function(x) data.frame(species = x$tip.label)), .id = 'clade') %>%
  mutate(clade = as.numeric(clade))

#same as above, but do this for the genetic tree time slices
jp.tree.taxa.80<-bind_rows(lapply(jp.tree.80, function(x) data.frame(species = x$tip.label)), .id = 'clade') %>%
  mutate(clade = as.numeric(clade))
jp.tree.taxa.100<-bind_rows(lapply(jp.tree.100, function(x) data.frame(species = x$tip.label)), .id = 'clade') %>%
  mutate(clade = as.numeric(clade))
jp.tree.taxa.120<-bind_rows(lapply(jp.tree.120, function(x) data.frame(species = x$tip.label)), .id = 'clade') %>%
  mutate(clade = as.numeric(clade))

#create an empty table to tabulate which clades from jp.tree match with those in the taxonomic clade
taxon.count.80<-data.frame(clade = 1:length(unique(jp.tree.taxa.80$clade)), jp.n = NA, dat.n = NA)
taxon.count.100<-data.frame(clade = 1:length(unique(jp.tree.taxa.100$clade)), jp.n = NA, dat.n = NA)
taxon.count.120<-data.frame(clade = 1:length(unique(jp.tree.taxa.120$clade)), jp.n = NA, dat.n = NA)

#loop through and identify a single taxon from each numbered clade of the taxonomic tree and match them to the numbered clades in jp.tree
for(i in 1:length(unique(jp.tree.taxa.80$clade))) {
  for(j in 1:length(unique(jp7k.taxa.80$clade))) {
    x.tmp<-jp.tree.taxa.80 %>%
      filter(clade == i) %>%
      pull(species)
    
    y.tmp<-jp7k.taxa.80 %>%
      filter(clade == j) %>%
      pull(species)
    
    if(sum(x.tmp %in% y.tmp)) {
      taxon.count.80[i, 'jp.n']<-length(y.tmp)
      taxon.count.80[i, 'dat.n']<-sum(sp.in.dat %in% x.tmp)
    }
  }
  rm(x.tmp, y.tmp)
}

for(i in 1:length(unique(jp.tree.taxa.100$clade))) {
  for(j in 1:length(unique(jp7k.taxa.100$clade))) {
    x.tmp<-jp.tree.taxa.100 %>%
      filter(clade == i) %>%
      pull(species)
    
    y.tmp<-jp7k.taxa.100 %>%
      filter(clade == j) %>%
      pull(species)
    
    if(sum(x.tmp %in% y.tmp)) {
      taxon.count.100[i, 'jp.n']<-length(y.tmp)
      taxon.count.100[i, 'dat.n']<-sum(sp.in.dat %in% x.tmp)
    }
  }
  rm(x.tmp, y.tmp)
}


for(i in 1:length(unique(jp.tree.taxa.120$clade))) {
  for(j in 1:length(unique(jp7k.taxa.120$clade))) {
    x.tmp<-jp.tree.taxa.120 %>%
      filter(clade == i) %>%
      pull(species)
    
    y.tmp<-jp7k.taxa.120 %>%
      filter(clade == j) %>%
      pull(species)
    
    if(sum(x.tmp %in% y.tmp)) {
      taxon.count.120[i, 'jp.n']<-length(y.tmp)
      taxon.count.120[i, 'dat.n']<-sum(sp.in.dat %in% x.tmp)
    }
  }
  rm(x.tmp, y.tmp)
}
```

Using the table above, we filtered each \(n\)-MY subtree that matched species
sampling in our morphological dataset and removed any subtrees that had
fewer than 2 species sampled. We similarly created a list of dataframes
that matched each list of trees.

```
tree.sub.80<-lapply(jp.tree.80[taxon.count.80 %>% filter(dat.n > 1) %>% pull(clade)], function(x) tryCatch({
  treedata(x, mean.ratio.dat %>% select(species, head.L:crus.v) %>% column_to_rownames('species'), sort = T, warnings = F)$phy},
  error = function(e) print('Check!')))
dat.80<-lapply(jp.tree.80[taxon.count.80 %>% filter(dat.n > 1) %>% pull(clade)], function(x) tryCatch({
  treedata(x, mean.ratio.dat %>% select(species, head.L:crus.v) %>% column_to_rownames('species'), sort = T, warnings = F)$data},
  error = function(e) print('Check!')))
tree.sub.100<-lapply(jp.tree.100[taxon.count.100 %>% filter(dat.n > 1) %>% pull(clade)], function(x) tryCatch({
  treedata(x, mean.ratio.dat %>% select(species, head.L:crus.v) %>% column_to_rownames('species'), sort = T, warnings = F)$phy},
  error = function(e) print('Check!')))
dat.100<-lapply(jp.tree.100[taxon.count.100 %>% filter(dat.n > 1) %>% pull(clade)], function(x) tryCatch({
  treedata(x, mean.ratio.dat %>% select(species, head.L:crus.v) %>% column_to_rownames('species'), sort = T, warnings = F)$data},
  error = function(e) print('Check!')))
tree.sub.120<-lapply(jp.tree.120[taxon.count.120 %>% filter(dat.n > 1) %>% pull(clade)], function(x) tryCatch({
  treedata(x, mean.ratio.dat %>% select(species, head.L:crus.v) %>% column_to_rownames('species'), sort = T, warnings = F)$phy},
  error = function(e) print('Check!')))
dat.120<-lapply(jp.tree.120[taxon.count.120 %>% filter(dat.n > 1) %>% pull(clade)], function(x) tryCatch({
  treedata(x, mean.ratio.dat %>% select(species, head.L:crus.v) %>% column_to_rownames('species'), sort = T, warnings = F)$data},
  error = function(e) print('Check!')))
```

Next, we used a custom function that matched clade numbers between
the two sets of subtrees. This was necessary because clade numbering is
arbitrary and the clade IDs here are simply numbered by the order in
which they appear. We then merged this table with the taxon-count tables
and removed any clades with less than 2 species.

```
match.clades<-function(jp, tax){
  tmp<-data.frame(taxa.clade = 1:length(unique(tax$clade)), jp.clade = NA)
  for(i in 1:length(unique(tax$clade))) {
    for(j in 1:length(unique(jp$clade))) {
      taxa.tmp<-tax %>%
        filter(clade == i) %>%
        pull(species)
      jp.tmp<-jp %>%
        filter(clade == j) %>%
        pull(species)
      if(sum(taxa.tmp %in% jp.tmp)) {
        tmp[i, 'jp.clade']<-j
      }
    }
  }
  tmp
}

match.80<-match.clades(jp7k.taxa.80, jp.tree.taxa.80) %>% rename('clade' = 'taxa.clade')
match.100<-match.clades(jp7k.taxa.100, jp.tree.taxa.100) %>% rename('clade' = 'taxa.clade')
match.120<-match.clades(jp7k.taxa.120, jp.tree.taxa.120) %>% rename('clade' = 'taxa.clade')

taxon.count.80<-taxon.count.80 %>%
  left_join(., match.80) %>%
  filter(dat.n > 1) %>%
  mutate(clade = as.numeric(rownames(.)))
```

```
## Joining with `by = join_by(clade)`
```

```
taxon.count.100<-taxon.count.100 %>%
  left_join(., match.100) %>%
  filter(dat.n > 1) %>%
  mutate(clade = as.numeric(rownames(.)))
```

```
## Joining with `by = join_by(clade)`
```

```
taxon.count.120<-taxon.count.120 %>%
  left_join(., match.120) %>%
  filter(dat.n > 1) %>%
  mutate(clade = as.numeric(rownames(.)))
```

```
## Joining with `by = join_by(clade)`
```

As we did before with the family-level dataset, we created a list of
vectors that grouped species together. All species were grouped as 1
because we were not concerned with groups, yet
`compare.evol.rates` requires a grouping object. We also
wrote a wrapper function for `compare.evol.rates` to work
with list objects.

```
gp.80<-gplist(dat.80)
gp.100<-gplist(dat.100)
gp.120<-gplist(dat.120)

sigmas.vec<-function(x, y, z) {
  mapply(function(x, y, z){
    compare.evol.rates(
      A = x,
      phy = y,
      gp = z,
      iter = 1
    )$sigma.d.all
  }, x = x,
  y = y,
  z = z)
}
```

Next we created a dataframe consisting of a single taxon from each
clade, then pruned the tree to match these taxa. This created a
clade-level tree where the tips were the numbered clades. We renamed the
tips to match the clade numbers and rotated the nodes.

```
tips.80<-bind_rows(lapply(dat.80, data.frame), .id = 'clade') %>%
  rownames_to_column('species') %>%
  select(species, clade) %>%
  group_by(clade) %>%
  slice_head(n = 1) %>%
  column_to_rownames('species')

clade.tree.80<-rotateNodes(treedata(jp.tree, tips.80, sort = T, warnings = F)$phy, 'all')
clade.tree.80$tip.label<-treedata(jp.tree, tips.80, sort = T, warnings = F)$data[,1]

tips.100<-bind_rows(lapply(dat.100, data.frame), .id = 'clade') %>%
  rownames_to_column('species') %>%
  select(species, clade) %>%
  group_by(clade) %>%
  slice_head(n = 1) %>%
  column_to_rownames('species')

clade.tree.100<-rotateNodes(treedata(jp.tree, tips.100, sort = T, warnings = F)$phy, 'all')
clade.tree.100$tip.label<-treedata(jp.tree, tips.100, sort = T, warnings = F)$data[,1]

tips.120<-bind_rows(lapply(dat.120, data.frame), .id = 'clade') %>%
  rownames_to_column('species') %>%
  arrange(desc(species)) %>%
  select(species, clade) %>%
  group_by(clade) %>%
  slice_head(n = 1) %>%
  column_to_rownames('species')

clade.tree.120<-rotateNodes(treedata(jp.tree, tips.120, sort = T, warnings = F)$phy, 'all')
clade.tree.120$tip.label<-treedata(jp.tree, tips.120, sort = T, warnings = F)$data[,1]
```

After that, we created a dataframe for each time slice with crown
age, stem age, and morphological rate. This dataframe was then merged
with species counts for each clade in each time slice. Then, using the
custom `crown.rate` and `stem.rate` functions, we
estimated net diversification rates for low, medium, and high extinction
fractions for crown and stem ages and bound them to each dataframe.

```
rates.80<-data.frame(crown.age = sapply(jp7k.80[taxon.count.80$jp.clade], function(x) max(nodeHeights(x))),
                     stem.age = clade.tree.80$edge.length[clade.tree.80$edge[,2] <= Ntip(clade.tree.80)],
                     ratio.sigma = sigmas.vec(x = dat.80, y = tree.sub.80, z = gp.80),
                     set = 80,
                     clade = 1:length(tree.sub.80)) %>%
  left_join(., taxon.count.80 %>% select(clade, jp.n, jp.clade), by = 'clade') %>%
  rename(n = jp.n)

rates.100<-data.frame(crown.age = sapply(jp7k.100[taxon.count.100$jp.clade], function(x) max(nodeHeights(x))),
                      stem.age = clade.tree.100$edge.length[clade.tree.100$edge[,2] <= Ntip(clade.tree.100)],
                      ratio.sigma = sigmas.vec(x = dat.100, y = tree.sub.100, z = gp.100),
                      set = 100,
                      clade = taxon.count.100$clade) %>%
  left_join(., taxon.count.100 %>% select(clade, jp.n, jp.clade), by = 'clade') %>%
  rename(n = jp.n)

rates.120<-data.frame(crown.age = sapply(jp7k.120[taxon.count.120$jp.clade], function(x) max(nodeHeights(x))),
                      stem.age = clade.tree.120$edge.length[clade.tree.120$edge[,2] <= Ntip(clade.tree.120)],
                      ratio.sigma = sigmas.vec(x = dat.120, y = tree.sub.120, z = gp.120),
                      set = 120,
                      clade = 1:length(tree.sub.120)) %>%
  left_join(., taxon.count.120 %>% select(clade, jp.n, jp.clade), by = 'clade') %>%
  rename(n = jp.n)


crown.rate<-function(z) {
  ext.rate <- c(0, 0.5, 0.9)
  names(ext.rate) <- c('low', 'med', 'high')
  crown.rates <- data.frame(lapply(ext.rate, function(x) {
    (log(
      0.5 * z$n * (1 - x ^ 2) +
        2 * x + 0.5 * (1 - x) * sqrt(
          z$n * (
            z$n * x ^ 2 - 8 * x + 2 * z$n * x + z$n
          )
        )
    ) - log(2)) / z$crown.age
  }))
  colnames(crown.rates) <-
    paste('crown', colnames(crown.rates), sep = '_')
  crown.rates
}

stem.rate<-function(z) {
  ext.rate<-c(0, 0.5, 0.9)
  names(ext.rate)<-c('low', 'med', 'high')
  stem.rates<-data.frame(lapply(ext.rate, function(x) {
    log(z$n * (1 - x) + x) / z$stem.age
  }))
  colnames(stem.rates)<-
    paste('stem', colnames(stem.rates), sep = '_')
  stem.rates
}

rates.80<-cbind(rates.80, crown.rate(rates.80), stem.rate(rates.80))
rates.100<-cbind(rates.100, crown.rate(rates.100), stem.rate(rates.100))
rates.120<-cbind(rates.120, crown.rate(rates.120), stem.rate(rates.120))
```

We then mean-centered and scaled each dataframe.

```
log.cs<-function(y) {
  y %>%
    mutate(across(crown_low:crown_high, ~ log(.x + 1)),
           across(c(ratio.sigma, stem_low:stem_high), ~ log(.x))) %>%
    relocate(ratio.sigma, .after = clade) %>%
    relocate(n, .after = clade) %>%
    mutate(across(ratio.sigma:stem_high, ~ scale(.x, center = T) %>% as.vector))
}

rates.80.cs<-log.cs(rates.80)
rates.100.cs<-log.cs(rates.100)
rates.120.cs<-log.cs(rates.120)
```

We then created a clade-identifier column for each time slice for
`ppca.scores2`.

```
clades.80<-bind_rows(lapply(dat.80, data.frame), .id ='dat.80') %>%
  rownames_to_column('species') %>%
  select(species,dat.80)

clades.120<-bind_rows(lapply(dat.120, data.frame), .id ='dat.120') %>%
  rownames_to_column('species') %>%
  select(species,dat.120)

clades.100<-bind_rows(lapply(dat.100, data.frame), .id ='dat.100') %>%
  rownames_to_column('species') %>%
  select(species,dat.100)

ppca.scores3<-ppca.scores2 %>%
  left_join(., clades.80) %>%
  left_join(., clades.100) %>%
  left_join(., clades.120)
```

```
## Joining with `by = join_by(species)`
## Joining with `by = join_by(species)`
## Joining with `by = join_by(species)`
```

Then we estimated convex hulls and hypervolumes for each time slice.
As above, the hypervolumes take some time to estimate, so we have
commented them out but provided them in the hypervolumes data file
loaded in the section “Quantifying
*n*-dimensional morphological diversity” above.

```
dat.80.ch<-list(
  get.vol(rates.80.cs, ppca.scores3, clades = 'clade', dataset = 'dat.80', column = 'crown_low', vol = 'conv'),
  get.vol(rates.80.cs, ppca.scores3, clades = 'clade', dataset = 'dat.80', column = 'crown_med', vol = 'conv'),
  get.vol(rates.80.cs, ppca.scores3, clades = 'clade', dataset = 'dat.80', column = 'crown_high', vol = 'conv'),
  get.vol(rates.80.cs, ppca.scores3, clades = 'clade', dataset = 'dat.80', column = 'stem_low', vol = 'conv'),
  get.vol(rates.80.cs, ppca.scores3, clades = 'clade', dataset = 'dat.80', column = 'stem_med', vol = 'conv'),
  get.vol(rates.80.cs, ppca.scores3, clades = 'clade', dataset = 'dat.80', column = 'stem_high', vol = 'conv')
)

dat.100.ch<-list(
  get.vol(rates.100.cs, ppca.scores3, clades = 'clade', dataset = 'dat.100', column = 'crown_low', vol = 'conv'),
  get.vol(rates.100.cs, ppca.scores3, clades = 'clade', dataset = 'dat.100', column = 'crown_med', vol = 'conv'),
  get.vol(rates.100.cs, ppca.scores3, clades = 'clade', dataset = 'dat.100', column = 'crown_high', vol = 'conv'),
  get.vol(rates.100.cs, ppca.scores3, clades = 'clade', dataset = 'dat.100', column = 'stem_low', vol = 'conv'),
  get.vol(rates.100.cs, ppca.scores3, clades = 'clade', dataset = 'dat.100', column = 'stem_med', vol = 'conv'),
  get.vol(rates.100.cs, ppca.scores3, clades = 'clade', dataset = 'dat.100', column = 'stem_high', vol = 'conv')
)

dat.120.ch<-list(
  get.vol(rates.120.cs, ppca.scores3, clades = 'clade', dataset = 'dat.120', column = 'crown_low', vol = 'conv'),
  get.vol(rates.120.cs, ppca.scores3, clades = 'clade', dataset = 'dat.120', column = 'crown_med', vol = 'conv'),
  get.vol(rates.120.cs, ppca.scores3, clades = 'clade', dataset = 'dat.120', column = 'crown_high', vol = 'conv'),
  get.vol(rates.120.cs, ppca.scores3, clades = 'clade', dataset = 'dat.120', column = 'stem_low', vol = 'conv'),
  get.vol(rates.120.cs, ppca.scores3, clades = 'clade', dataset = 'dat.120', column = 'stem_med', vol = 'conv'),
  get.vol(rates.120.cs, ppca.scores3, clades = 'clade', dataset = 'dat.120', column = 'stem_high', vol = 'conv')
)

# system.time(dat.80.hv<-list(
#   get.vol(rates.80.cs, ppca.scores3, clades = 'clade', dataset = 'dat.80', column = 'crown_low', vol = 'hyper'),
#   get.vol(rates.80.cs, ppca.scores3, clades = 'clade', dataset = 'dat.80', column = 'crown_med', vol = 'hyper'),
#   get.vol(rates.80.cs, ppca.scores3, clades = 'clade', dataset = 'dat.80', column = 'crown_high', vol = 'hyper'),
#   get.vol(rates.80.cs, ppca.scores3, clades = 'clade', dataset = 'dat.80', column = 'stem_low', vol = 'hyper'),
#   get.vol(rates.80.cs, ppca.scores3, clades = 'clade', dataset = 'dat.80', column = 'stem_med', vol = 'hyper'),
#   get.vol(rates.80.cs, ppca.scores3, clades = 'clade', dataset = 'dat.80', column = 'stem_high', vol = 'hyper')
# )) ### took  1461 seconds
# 
# system.time(dat.100.hv<-list(
#   get.vol(rates.100.cs, ppca.scores3, clades = 'clade', dataset = 'dat.100', column = 'crown_low', vol = 'hyper'),
#   get.vol(rates.100.cs, ppca.scores3, clades = 'clade', dataset = 'dat.100', column = 'crown_med', vol = 'hyper'),
#   get.vol(rates.100.cs, ppca.scores3, clades = 'clade', dataset = 'dat.100', column = 'crown_high', vol = 'hyper'),
#   get.vol(rates.100.cs, ppca.scores3, clades = 'clade', dataset = 'dat.100', column = 'stem_low', vol = 'hyper'),
#   get.vol(rates.100.cs, ppca.scores3, clades = 'clade', dataset = 'dat.100', column = 'stem_med', vol = 'hyper'),
#   get.vol(rates.100.cs, ppca.scores3, clades = 'clade', dataset = 'dat.100', column = 'stem_high', vol = 'hyper')
# )) ### took 2517 seconds
# 
# system.time(dat.120.hv<-list(
#   get.vol(rates.120.cs, ppca.scores3, clades = 'clade', dataset = 'dat.120', column = 'crown_low', vol = 'hyper'),
#   get.vol(rates.120.cs, ppca.scores3, clades = 'clade', dataset = 'dat.120', column = 'crown_med', vol = 'hyper'),
#   get.vol(rates.120.cs, ppca.scores3, clades = 'clade', dataset = 'dat.120', column = 'crown_high', vol = 'hyper'),
#   get.vol(rates.120.cs, ppca.scores3, clades = 'clade', dataset = 'dat.120', column = 'stem_low', vol = 'hyper'),
#   get.vol(rates.120.cs, ppca.scores3, clades = 'clade', dataset = 'dat.120', column = 'stem_med', vol = 'hyper'),
#   get.vol(rates.120.cs, ppca.scores3, clades = 'clade', dataset = 'dat.120', column = 'stem_high', vol = 'hyper')
# )) ### took 1622 seconds
```

We next created tables of volume and species-richness proportions for
each quadrant for time-sliced clades.

```
dat.80.ch.table<-bind_rows(lapply(dat.80.ch, function(x) x$table))
dat.100.ch.table<-bind_rows(lapply(dat.100.ch, function(x) x$table))
dat.120.ch.table<-bind_rows(lapply(dat.120.ch, function(x) x$table))

# dat.80.hv.table<-bind_rows(lapply(dat.80.hv, function(x) x$table))
# dat.100.hv.table<-bind_rows(lapply(dat.100.hv, function(x) x$table))
# dat.120.hv.table<-bind_rows(lapply(dat.120.hv, function(x) x$table))

dat.80.ch.table$vol.prop<-dat.80.ch.table$vols/frog.ch$vol
dat.100.ch.table$vol.prop<-dat.100.ch.table$vols/frog.ch$vol
dat.120.ch.table$vol.prop<-dat.120.ch.table$vols/frog.ch$vol
dat.80.ch.table$n.prop<-dat.80.ch.table$n/(dat.80.ch.table %>% 
                                                 group_by(div.rate) %>%
                                                 summarise(tot = sum(n)) %>%
                                                 pull(tot) %>%
                                                 unique())
dat.100.ch.table$n.prop<-dat.100.ch.table$n/(dat.100.ch.table %>% 
                                                   group_by(div.rate) %>%
                                                   summarise(tot = sum(n)) %>%
                                                   pull(tot) %>%
                                                   unique())
dat.120.ch.table$n.prop<-dat.120.ch.table$n/(dat.120.ch.table %>% 
                                                   group_by(div.rate) %>%
                                                   summarise(tot = sum(n)) %>%
                                                   pull(tot) %>%
                                                   unique())
# dat.80.hv.table$vol.prop<-dat.80.hv.table$vols/get_volume(frog.hv)
# dat.100.hv.table$vol.prop<-dat.100.hv.table$vols/get_volume(frog.hv)
# dat.120.hv.table$vol.prop<-dat.120.hv.table$vols/get_volume(frog.hv)
```

Next we estimated hypervolumes and convex hulls, but this time using
medians as quadrant boundaries for each time slice. As with above, all
hypervolume code is commented out because it takes a long time to
run.

```
dat.80.med.ch<-list(
  get.vol(rates.80.cs, ppca.scores3, clades = 'clade', dataset = 'dat.80', column = 'crown_low', vol = 'conv', med =T),
  get.vol(rates.80.cs, ppca.scores3, clades = 'clade', dataset = 'dat.80', column = 'crown_med', vol = 'conv', med =T),
  get.vol(rates.80.cs, ppca.scores3, clades = 'clade', dataset = 'dat.80', column = 'crown_high', vol = 'conv', med =T),
  get.vol(rates.80.cs, ppca.scores3, clades = 'clade', dataset = 'dat.80', column = 'stem_low', vol = 'conv', med =T),
  get.vol(rates.80.cs, ppca.scores3, clades = 'clade', dataset = 'dat.80', column = 'stem_med', vol = 'conv', med =T),
  get.vol(rates.80.cs, ppca.scores3, clades = 'clade', dataset = 'dat.80', column = 'stem_high', vol = 'conv', med =T)
)

dat.100.med.ch<-list(
  get.vol(rates.100.cs, ppca.scores3, clades = 'clade', dataset = 'dat.100', column = 'crown_low', vol = 'conv', med =T),
  get.vol(rates.100.cs, ppca.scores3, clades = 'clade', dataset = 'dat.100', column = 'crown_med', vol = 'conv', med =T),
  get.vol(rates.100.cs, ppca.scores3, clades = 'clade', dataset = 'dat.100', column = 'crown_high', vol = 'conv', med =T),
  get.vol(rates.100.cs, ppca.scores3, clades = 'clade', dataset = 'dat.100', column = 'stem_low', vol = 'conv', med =T),
  get.vol(rates.100.cs, ppca.scores3, clades = 'clade', dataset = 'dat.100', column = 'stem_med', vol = 'conv', med =T),
  get.vol(rates.100.cs, ppca.scores3, clades = 'clade', dataset = 'dat.100', column = 'stem_high', vol = 'conv', med =T)
)

dat.120.med.ch<-list(
  get.vol(rates.120.cs, ppca.scores3, clades = 'clade', dataset = 'dat.120', column = 'crown_low', vol = 'conv', med =T),
  get.vol(rates.120.cs, ppca.scores3, clades = 'clade', dataset = 'dat.120', column = 'crown_med', vol = 'conv', med =T),
  get.vol(rates.120.cs, ppca.scores3, clades = 'clade', dataset = 'dat.120', column = 'crown_high', vol = 'conv', med =T),
  get.vol(rates.120.cs, ppca.scores3, clades = 'clade', dataset = 'dat.120', column = 'stem_low', vol = 'conv', med =T),
  get.vol(rates.120.cs, ppca.scores3, clades = 'clade', dataset = 'dat.120', column = 'stem_med', vol = 'conv', med =T),
  get.vol(rates.120.cs, ppca.scores3, clades = 'clade', dataset = 'dat.120', column = 'stem_high', vol = 'conv', med =T)
)

# system.time(dat.80.med.hv<-list(
#   get.vol(rates.80.cs, ppca.scores3, clades = 'clade', dataset = 'dat.80', column = 'crown_low', vol = 'hyper', med =T),
#   get.vol(rates.80.cs, ppca.scores3, clades = 'clade', dataset = 'dat.80', column = 'crown_med', vol = 'hyper', med =T),
#   get.vol(rates.80.cs, ppca.scores3, clades = 'clade', dataset = 'dat.80', column = 'crown_high', vol = 'hyper', med =T),
#   get.vol(rates.80.cs, ppca.scores3, clades = 'clade', dataset = 'dat.80', column = 'stem_low', vol = 'hyper', med =T),
#   get.vol(rates.80.cs, ppca.scores3, clades = 'clade', dataset = 'dat.80', column = 'stem_med', vol = 'hyper', med =T),
#   get.vol(rates.80.cs, ppca.scores3, clades = 'clade', dataset = 'dat.80', column = 'stem_high', vol = 'hyper', med =T)
# )) ### took 1346 seconds
# 
# system.time(dat.100.med.hv<-list(
#   get.vol(rates.100.cs, ppca.scores3, clades = 'clade', dataset = 'dat.100', column = 'crown_low', vol = 'hyper', med =T),
#   get.vol(rates.100.cs, ppca.scores3, clades = 'clade', dataset = 'dat.100', column = 'crown_med', vol = 'hyper', med =T),
#   get.vol(rates.100.cs, ppca.scores3, clades = 'clade', dataset = 'dat.100', column = 'crown_high', vol = 'hyper', med =T),
#   get.vol(rates.100.cs, ppca.scores3, clades = 'clade', dataset = 'dat.100', column = 'stem_low', vol = 'hyper', med =T),
#   get.vol(rates.100.cs, ppca.scores3, clades = 'clade', dataset = 'dat.100', column = 'stem_med', vol = 'hyper', med =T),
#   get.vol(rates.100.cs, ppca.scores3, clades = 'clade', dataset = 'dat.100', column = 'stem_high', vol = 'hyper', med =T)
# )) ### took 1552 seconds
# 
# system.time(dat.120.med.hv<-list(
#   get.vol(rates.120.cs, ppca.scores3, clades = 'clade', dataset = 'dat.120', column = 'crown_low', vol = 'hyper', med =T),
#   get.vol(rates.120.cs, ppca.scores3, clades = 'clade', dataset = 'dat.120', column = 'crown_med', vol = 'hyper', med =T),
#   get.vol(rates.120.cs, ppca.scores3, clades = 'clade', dataset = 'dat.120', column = 'crown_high', vol = 'hyper', med =T),
#   get.vol(rates.120.cs, ppca.scores3, clades = 'clade', dataset = 'dat.120', column = 'stem_low', vol = 'hyper', med =T),
#   get.vol(rates.120.cs, ppca.scores3, clades = 'clade', dataset = 'dat.120', column = 'stem_med', vol = 'hyper', med =T),
#   get.vol(rates.120.cs, ppca.scores3, clades = 'clade', dataset = 'dat.120', column = 'stem_high', vol = 'hyper', med =T)
# )) ### took seconds
```

We also created tables for the median-based hypervolumes and convex
hulls.

```
dat.80.med.ch.table<-bind_rows(lapply(dat.80.med.ch, function(x) x$table))
dat.100.med.ch.table<-bind_rows(lapply(dat.100.med.ch, function(x) x$table))
dat.120.med.ch.table<-bind_rows(lapply(dat.120.med.ch, function(x) x$table))

# dat.80.med.hv.table<-bind_rows(lapply(dat.80.med.hv, function(x) x$table))
# dat.100.med.hv.table<-bind_rows(lapply(dat.100.med.hv, function(x) x$table))
# dat.120.med.hv.table<-bind_rows(lapply(dat.120.med.hv, function(x) x$table))

dat.80.med.ch.table$vol.prop<-dat.80.med.ch.table$vols/frog.ch$vol
dat.100.med.ch.table$vol.prop<-dat.100.med.ch.table$vols/frog.ch$vol
dat.120.med.ch.table$vol.prop<-dat.120.med.ch.table$vols/frog.ch$vol
dat.80.med.ch.table$n.prop<-dat.80.med.ch.table$n/(dat.80.med.ch.table %>%
                                                         group_by(div.rate) %>%
                                                         summarise(n.tot = sum(n)) %>%
                                                         pull(n.tot) %>%
                                                         unique())
dat.100.med.ch.table$n.prop<-dat.100.med.ch.table$n/(dat.100.med.ch.table %>%
                                                           group_by(div.rate) %>%
                                                           summarise(n.tot = sum(n)) %>%
                                                           pull(n.tot) %>%
                                                           unique())
dat.120.med.ch.table$n.prop<-dat.120.med.ch.table$n/(dat.120.med.ch.table %>%
                                                           group_by(div.rate) %>%
                                                           summarise(n.tot = sum(n)) %>%
                                                           pull(n.tot) %>%
                                                           unique())
# dat.80.med.hv.table$vol.prop<-dat.80.med.hv.table$vols/get_volume(frog.hv)
# dat.100.med.hv.table$vol.prop<-dat.100.med.hv.table$vols/get_volume(frog.hv)
# dat.120.med.hv.table$vol.prop<-dat.120.med.hv.table$vols/get_volume(frog.hv)
```

To summarize these results, we created Supplementary Figure 4. Note
that our figure annotations are not presented here. However, the top row
shows 80 MY slices, the middle row shows 100 MY slices, and the bottom
row shows 120 MY slices. Here we only show net diversification rates
estimated using stem ages and moderate extinction rates, as in our
primary analyses.

```
(median_mean_timeslice_plot<-ggplot(rates.80.cs, aes(x = stem_med, y = ratio.sigma)) +
  geom_hline(yintercept = median(rates.80.cs$ratio.sigma)) +
  geom_vline(xintercept = median(rates.80.cs$stem_med)) +
  geom_point(size = 3, shape = 21, fill = 'grey') +
  scale_x_continuous(breaks = pretty_breaks(6), limits = c(-3, 3)) +
  scale_y_continuous(breaks = pretty_breaks(6), limits = c(-3, 3)) +
  theme_bw() +
  ggtitle('medians') +
  theme(aspect.ratio = 1,
        axis.title.y = element_blank(),
        axis.text.x = element_blank(),
        axis.title.x = element_blank()) +
ggplot(rates.100.cs, aes(x = stem_med, y = ratio.sigma)) +
  geom_hline(yintercept = median(rates.100.cs$ratio.sigma)) +
  geom_vline(xintercept = median(rates.100.cs$stem_med)) +
  geom_point(size = 3, shape = 21, fill = 'grey') +
  scale_x_continuous(breaks = pretty_breaks(6), limits = c(-3, 3)) +
  scale_y_continuous(breaks = pretty_breaks(6), limits = c(-3, 3),
                     expression(paste('Morphological Rate ', (sigma^{2})))) +
  theme_bw() +
  theme(aspect.ratio = 1,
        axis.title.x = element_blank(),
        axis.text.x = element_blank()) +
ggplot(rates.120.cs, aes(x = stem_med, y = ratio.sigma)) +
  geom_hline(yintercept = median(rates.120.cs$ratio.sigma)) +
  geom_vline(xintercept = median(rates.120.cs$stem_med)) +
  geom_point(size = 3, shape = 21, fill = 'grey') +
  scale_x_continuous('Net diversification rate', breaks = pretty_breaks(6), limits = c(-3, 3)) +
  scale_y_continuous(breaks = pretty_breaks(6), limits = c(-3, 3)) +
  theme_bw() +
  theme(aspect.ratio = 1,
        axis.title.y = element_blank()) +
ggplot(rates.80.cs, aes(x = stem_med, y = ratio.sigma)) +
  geom_hline(yintercept = 0) +
  geom_vline(xintercept = 0) +
  geom_point(size = 3, shape = 21, fill = 'grey') +
  scale_x_continuous(breaks = pretty_breaks(6), limits = c(-3, 3)) +
  scale_y_continuous(breaks = pretty_breaks(6), limits = c(-3, 3)) +
  ggtitle('means') +
  theme_bw() +
  theme(aspect.ratio = 1,
        axis.title.y = element_blank(),
        axis.text.y = element_blank(),
        axis.text.x = element_blank(),
        axis.title.x = element_blank()) +
ggplot(rates.100.cs, aes(x = stem_med, y = ratio.sigma)) +
  geom_hline(yintercept = 0) +
  geom_vline(xintercept = 0) +
  geom_point(size = 3, shape = 21, fill = 'grey') +
  scale_x_continuous(breaks = pretty_breaks(6), limits = c(-3, 3)) +
  scale_y_continuous(breaks = pretty_breaks(6), limits = c(-3, 3)) +
  theme_bw() +
  theme(aspect.ratio = 1,
        axis.title.y = element_blank(),
        axis.text.y = element_blank(),
        axis.text.x = element_blank(),
        axis.title.x = element_blank()) +
ggplot(rates.120.cs, aes(x = stem_med, y = ratio.sigma)) +
  geom_hline(yintercept = 0) +
  geom_vline(xintercept = 0) +
  geom_point(size = 3, shape = 21, fill = 'grey') +
  scale_x_continuous('Net diversification rate', breaks = pretty_breaks(6), limits = c(-3, 3)) +
  scale_y_continuous(breaks = pretty_breaks(6), limits = c(-3, 3)) +
  theme_bw() +
  theme(aspect.ratio = 1,
        axis.title.y = element_blank(),
        axis.text.y = element_blank()) +
  plot_layout(ncol = 2, nrow = 3, byrow = F))
```

```
#convex hull proportions boundaries defined by medians
bind_rows('80 MYA slice' = dat.80.med.ch.table %>% filter(div.rate == 'stem_med'),
          '100 MYA slice' = dat.100.med.ch.table %>% filter(div.rate == 'stem_med'),
          '120 MYA slice' = dat.120.med.ch.table %>% filter(div.rate == 'stem_med'), .id = 'slice')
```

```
##                   slice quad        vols      n div.rate     vol.prop
## ar...1     80 MYA slice   AR 2.879505578 3860.5 stem_med 0.6683548306
## anr...2    80 MYA slice  ANR 0.721762387  376.0 stem_med 0.1675264607
## nanr...3   80 MYA slice NANR 0.464596296  334.5 stem_med 0.1078362830
## nar...4    80 MYA slice  NAR 0.723419773 1617.0 stem_med 0.1679111523
## ar...5    100 MYA slice   AR 2.969798971 3421.0 stem_med 0.6893126039
## anr...6   100 MYA slice  ANR 0.530066675  466.0 stem_med 0.1230324488
## nanr...7  100 MYA slice NANR 0.072892019   57.0 stem_med 0.0169187839
## nar...8   100 MYA slice  NAR 1.194659532 1389.0 stem_med 0.2772894331
## ar...9    120 MYA slice   AR 3.837036606 5452.0 stem_med 0.8906049600
## anr...10  120 MYA slice  ANR 0.002512618   19.0 stem_med 0.0005831974
## nanr...11 120 MYA slice NANR 0.028109223   38.0 stem_med 0.0065243614
## nar...12  120 MYA slice  NAR 0.744668367  779.0 stem_med 0.1728431103
##                n.prop
## ar...1    0.623868778
## anr...2   0.060762767
## nanr...3  0.054056238
## nar...4   0.261312217
## ar...5    0.641477592
## anr...6   0.087380461
## nanr...7  0.010688168
## nar...8   0.260453778
## ar...9    0.867048346
## anr...10  0.003021628
## nanr...11 0.006043257
## nar...12  0.123886768
```

```
#hypervolume proportions boundaries defined by medians
bind_rows('80 MYA slice' = dat.80.ch.table %>% filter(div.rate == 'stem_med'),
          '100 MYA slice' = dat.100.ch.table %>% filter(div.rate == 'stem_med'),
          '120 MYA slice' = dat.120.ch.table %>% filter(div.rate == 'stem_med'), .id = 'slice')
```

```
##                   slice quad         vols    n div.rate     vol.prop
## ar...1     80 MYA slice   AR 3.8658471721 5232 stem_med 8.972921e-01
## anr...2    80 MYA slice  ANR 0.3222191753  343 stem_med 7.478949e-02
## nanr...3   80 MYA slice NANR 0.2656373599   90 stem_med 6.165642e-02
## nar...4    80 MYA slice  NAR 0.0771079353  523 stem_med 1.789733e-02
## ar...5    100 MYA slice   AR 4.2000006435 5187 stem_med 9.748516e-01
## anr...6   100 MYA slice  ANR 0.0129002252   41 stem_med 2.994239e-03
## nanr...7  100 MYA slice NANR 0.0728920185   57 stem_med 1.691878e-02
## nar...8   100 MYA slice  NAR 0.0002294126   48 stem_med 5.324838e-05
## ar...9    120 MYA slice   AR 4.2168988123 6174 stem_med 9.787738e-01
## anr...10  120 MYA slice  ANR 0.0025126176   19 stem_med 5.831974e-04
## nanr...11 120 MYA slice NANR 0.0347173448   43 stem_med 8.058156e-03
## nar...12  120 MYA slice  NAR 0.0005562145   52 stem_med 1.291015e-04
##                n.prop
## ar...1    0.845507434
## anr...2   0.055429864
## nanr...3  0.014544279
## nar...4   0.084518423
## ar...5    0.972623289
## anr...6   0.007687980
## nanr...7  0.010688168
## nar...8   0.009000563
## ar...9    0.981870229
## anr...10  0.003021628
## nanr...11 0.006838422
## nar...12  0.008269720
```

```
#convex hull proportions boundaries defined by means
bind_rows('80 MYA slice' = dat.80.ch.table %>% filter(div.rate == 'stem_med'),
          '100 MYA slice' = dat.100.ch.table %>% filter(div.rate == 'stem_med'),
          '120 MYA slice' = dat.120.ch.table %>% filter(div.rate == 'stem_med'), .id = 'slice')
```

```
##                   slice quad         vols    n div.rate     vol.prop
## ar...1     80 MYA slice   AR 3.8658471721 5232 stem_med 8.972921e-01
## anr...2    80 MYA slice  ANR 0.3222191753  343 stem_med 7.478949e-02
## nanr...3   80 MYA slice NANR 0.2656373599   90 stem_med 6.165642e-02
## nar...4    80 MYA slice  NAR 0.0771079353  523 stem_med 1.789733e-02
## ar...5    100 MYA slice   AR 4.2000006435 5187 stem_med 9.748516e-01
## anr...6   100 MYA slice  ANR 0.0129002252   41 stem_med 2.994239e-03
## nanr...7  100 MYA slice NANR 0.0728920185   57 stem_med 1.691878e-02
## nar...8   100 MYA slice  NAR 0.0002294126   48 stem_med 5.324838e-05
## ar...9    120 MYA slice   AR 4.2168988123 6174 stem_med 9.787738e-01
## anr...10  120 MYA slice  ANR 0.0025126176   19 stem_med 5.831974e-04
## nanr...11 120 MYA slice NANR 0.0347173448   43 stem_med 8.058156e-03
## nar...12  120 MYA slice  NAR 0.0005562145   52 stem_med 1.291015e-04
##                n.prop
## ar...1    0.845507434
## anr...2   0.055429864
## nanr...3  0.014544279
## nar...4   0.084518423
## ar...5    0.972623289
## anr...6   0.007687980
## nanr...7  0.010688168
## nar...8   0.009000563
## ar...9    0.981870229
## anr...10  0.003021628
## nanr...11 0.006838422
## nar...12  0.008269720
```

```
#hypervolume proportions boundaries defined by means
bind_rows('80 MYA slice' = dat.80.ch.table %>% filter(div.rate == 'stem_med'),
          '100 MYA slice' = dat.100.ch.table %>% filter(div.rate == 'stem_med'),
          '120 MYA slice' = dat.120.ch.table %>% filter(div.rate == 'stem_med'), .id = 'slice')
```

```
##                   slice quad         vols    n div.rate     vol.prop
## ar...1     80 MYA slice   AR 3.8658471721 5232 stem_med 8.972921e-01
## anr...2    80 MYA slice  ANR 0.3222191753  343 stem_med 7.478949e-02
## nanr...3   80 MYA slice NANR 0.2656373599   90 stem_med 6.165642e-02
## nar...4    80 MYA slice  NAR 0.0771079353  523 stem_med 1.789733e-02
## ar...5    100 MYA slice   AR 4.2000006435 5187 stem_med 9.748516e-01
## anr...6   100 MYA slice  ANR 0.0129002252   41 stem_med 2.994239e-03
## nanr...7  100 MYA slice NANR 0.0728920185   57 stem_med 1.691878e-02
## nar...8   100 MYA slice  NAR 0.0002294126   48 stem_med 5.324838e-05
## ar...9    120 MYA slice   AR 4.2168988123 6174 stem_med 9.787738e-01
## anr...10  120 MYA slice  ANR 0.0025126176   19 stem_med 5.831974e-04
## nanr...11 120 MYA slice NANR 0.0347173448   43 stem_med 8.058156e-03
## nar...12  120 MYA slice  NAR 0.0005562145   52 stem_med 1.291015e-04
##                n.prop
## ar...1    0.845507434
## anr...2   0.055429864
## nanr...3  0.014544279
## nar...4   0.084518423
## ar...5    0.972623289
## anr...6   0.007687980
## nanr...7  0.010688168
## nar...8   0.009000563
## ar...9    0.981870229
## anr...10  0.003021628
## nanr...11 0.006838422
## nar...12  0.008269720
```

Lastly, we also estimated convex hulls and hypervolumes using the
first four and six pPCA axes. We did this to test the sensitivity of our
results to estimating these morphospaces with five pPCA axes \*i.e., in
our primary results). We used modified versions of `get.vol`
to handle four and six dimensions. These results constituted
Supplementary Table 4.

```
frog4d.ch<-convhulln(p = ppca.scores2[,2:5], 'FA')
frog6d.ch<-convhulln(p = ppca.scores2[,2:7], 'FA')
# frog4d.hv<-hypervolume_svm(data = ppca.scores2[,2:5])  ###this was quick...
# system.time(frog6d.hv<-hypervolume_svm(data = ppca.scores2[,2:7])) ###took 463 seconds
quads.4d.ch<-get.vol.4d(rates = fam.dat.cs, scores = ppca.scores2, clades = 'family', dataset = 'family', column = 'stem_med', vol = 'conv')
quads.6d.ch<-get.vol.6d(rates = fam.dat.cs, scores = ppca.scores2, clades = 'family', dataset = 'family', column = 'stem_med', vol = 'conv')

quads.4d.ch.table<-quads.4d.ch$table
quads.4d.ch.table$vol.prop<-quads.4d.ch.table$vol/frog4d.ch$vol

quads.6d.ch.table<-quads.6d.ch$table
quads.6d.ch.table$vol.prop<-quads.6d.ch.table$vol/frog6d.ch$vol
# system.time(quads.4d.hv<-get.vol.4d(rates = fam.dat.cs, scores = ppca.scores2, clades = 'family', dataset = 'family', column = 'stem_med', vol = 'hyper')
#   # get.vol(rates = fam.dat.cs, scores = ppca.scores2, clades = 'family', dataset = 'family',column = 'bd_rates', vol = 'hyper')
# ) ####took 37 seconds
# quads.4d.hv.table<-quads.4d.hv$table
# quads.4d.hv.table$vol.prop<-quads.4d.hv.table$vol/get_volume(frog4d.hv)


# system.time(quads.6d.hv<-get.vol.6d(rates = fam.dat.cs, scores = ppca.scores2, clades = 'family', dataset = 'family', column = 'stem_med', vol = 'hyper')
#             # get.vol(rates = fam.dat.cs, scores = ppca.scores2, clades = 'family', dataset = 'family',column = 'bd_rates', vol = 'hyper')
# ) ####took 2312 seconds
# quads.6d.hv.table<-quads.6d.hv$table
# quads.6d.hv.table$vol.prop<-quads.6d.hv.table$vol/get_volume(frog6d.hv)

# Convex-hull results
left_join(quads.4d.ch.table %>% select(quad, vol.prop) %>% rename(four.d = vol.prop), quads.ch.table %>% filter(div.rate == 'stem_med') %>% select(quad, vol.prop) %>% rename(five.d = vol.prop)) %>%
  left_join(quads.6d.ch.table %>% select(quad, vol.prop) %>% rename(six.d = vol.prop))
```

```
## Joining with `by = join_by(quad)`
## Joining with `by = join_by(quad)`
```

```
##   quad     four.d     five.d      six.d
## 1   AR 0.75689213 0.66627912 0.51284537
## 2  ANR 0.21062831 0.11835216 0.08155462
## 3 NANR 0.05820006 0.02384559 0.01021939
## 4  NAR 0.26749183 0.13492440 0.06543735
```

```
# Hypervolume results
left_join(quads.4d.hv.table %>% select(quad, vol.prop) %>% filter() %>% rename(four.d = vol.prop), quads.hv.table %>% filter(div.rate == 'stem_med') %>% select(quad, vol.prop) %>% rename(five.d = vol.prop)) %>%
  left_join(quads.6d.hv.table %>% select(quad, vol.prop) %>% rename(six.d = vol.prop))
```

```
## Joining with `by = join_by(quad)`
## Joining with `by = join_by(quad)`
```

```
##   quad     four.d     five.d      six.d
## 1   AR 0.74690377 0.75428048 0.73916486
## 2  ANR 0.24541346 0.15477959 0.12375133
## 3 NANR 0.05197851 0.02583919 0.01501743
## 4  NAR 0.26357256 0.14125447 0.09386476
```

## Time dependence of net diversification rates and rates of morphological evolution

As a supplementary analysis for our discussion, we explored the
potential effects of time-dependence on the rates we estimated. We
circumscribed these analyses to include only stem ages at moderate
extinction rates (\(\varepsilon\) =
0.5) and the morphological rates of evolution. We first examined the
time dependence by regressing each of the mean-centered and scaled rates
by clade age. For net diversification rates, we regressed against stem
age, as this age was the most directly relevant (i.e., that used for
estimating the rates). By contrast, we used crown ages for regressing
rates of morphological evolution on time, since only crown phylogenies
are used for estimating the rates.

We estimated regression relationships using the
`phylolm::phylolm` function, which allowed us to perform
phylogenetic generalized least squares. We also plotted these
relationships, as in Supplementary Figure 5.

```
ratio.sigma.lm<-phylolm(ratio.sigma ~ crown.age, data = fam.dat.cs %>% column_to_rownames('family'),
                        phy = treedata(fam.tree, fam.dat.cs %>% column_to_rownames('family'), sort = T, warning = F)$phy,
                        model = 'BM')

stem_med.lm<-phylolm(stem_med ~ stem.age, data = fam.dat.cs %>% column_to_rownames('family'),
                     phy = treedata(fam.tree, fam.dat.cs %>% column_to_rownames('family'), sort = T, warning = F)$phy,
                     model = 'BM')

summary(ratio.sigma.lm)
```

```
## 
## Call:
## phylolm(formula = ratio.sigma ~ crown.age, data = fam.dat.cs %>% 
##     column_to_rownames("family"), phy = treedata(fam.tree, fam.dat.cs %>% 
##     column_to_rownames("family"), sort = T, warning = F)$phy, 
##     model = "BM")
## 
##    AIC logLik 
## 129.45 -61.73 
## 
## Raw residuals:
##     Min      1Q  Median      3Q     Max 
## -2.4516 -0.1241  0.3086  0.5903  2.6940 
## 
## Mean tip height: 221.2011
## Parameter estimate(s) using ML:
## sigma2: 0.007202883 
## 
## Coefficients:
##               Estimate     StdErr t.value p.value  
## (Intercept) -1.2302143  0.7094648 -1.7340 0.09043 .
## crown.age    0.0137549  0.0064787  2.1231 0.03983 *
## ---
## Signif. codes:  0 '***' 0.001 '**' 0.01 '*' 0.05 '.' 0.1 ' ' 1
## 
## R-squared: 0.09905   Adjusted R-squared: 0.07708
```

```
summary(stem_med.lm)
```

```
## 
## Call:
## phylolm(formula = stem_med ~ stem.age, data = fam.dat.cs %>% 
##     column_to_rownames("family"), phy = treedata(fam.tree, fam.dat.cs %>% 
##     column_to_rownames("family"), sort = T, warning = F)$phy, 
##     model = "BM")
## 
##    AIC logLik 
##  90.67 -42.33 
## 
## Raw residuals:
##      Min       1Q   Median       3Q      Max 
## -1.82017  0.03614  0.61314  0.89849  1.48733 
## 
## Mean tip height: 221.2011
## Parameter estimate(s) using ML:
## sigma2: 0.002922603 
## 
## Coefficients:
##              Estimate    StdErr t.value  p.value    
## (Intercept)  1.743848  0.824107  2.1160 0.040459 *  
## stem.age    -0.020017  0.005419 -3.6938 0.000646 ***
## ---
## Signif. codes:  0 '***' 0.001 '**' 0.01 '*' 0.05 '.' 0.1 ' ' 1
## 
## R-squared: 0.2497    Adjusted R-squared: 0.2314
```

```
ratio.lm.plot<-ggplot(fam.dat.cs, aes(x = crown.age, y = ratio.sigma)) +
  geom_point(size = 2, fill = 'grey', shape = 21) +
  geom_line(data = data.frame(ratio.sigma.lm$X, ratio.sigma = predict(ratio.sigma.lm)),
            aes(x = crown.age, y = ratio.sigma), color = 'black', lwd = 1) +
  ggtitle('a.') +
  labs(y = expression(paste('Morphological Rate ', (sigma^{2}))),
       x = 'Crown age')+
  theme_bw() +
  theme(aspect.ratio = 1,
        axis.text = element_text(color = 'black'))

stem_med.lm.plot<-ggplot(fam.dat.cs, aes(x = stem.age, y = stem_med)) +
  geom_point(size = 2, fill = 'grey', shape = 21) +
  geom_line(data = data.frame(stem_med.lm$X, stem_med = predict(stem_med.lm)),
            aes(x = stem.age, y = stem_med), color = 'black', lwd = 1) +
  labs(y = 'Net diversification rate (stem ages, moderate extinction fraction)',
       x = 'Stem age') +
  ggtitle('b.') +
  theme_bw() +
  theme(aspect.ratio = 1,
        axis.text = element_text(color = 'black'))

(r.rates.plot<-ratio.lm.plot + stem_med.lm.plot)
```

Here, we found that rates of both net diversification and
morphological evolution are significantly related to their respective
measures of clade age. We next calculated residuals from each linear
model to generate time-independent rates. We then used pgls to calculate
the correlation between time-independent measures of net diversification
rates and rates of morphological evolution, finding no correlation.

```
r.fam.dat<-data.frame(r.stem_med = resid(stem_med.lm), r.ratio.sigma = resid(ratio.sigma.lm))
(r.rates.corr<-gls.corr(dat = r.fam.dat,
                       tree = treedata(phy = fam.tree, data = r.fam.dat, sort = T, warnings = F)$phy)$unbiased.cors)
```

```
##               r.stem_med r.ratio.sigma
## r.stem_med    1.00000000    0.03480093
## r.ratio.sigma 0.03480093    1.00000000
```

```
cor.pvalue(r.rates.corr[2,1], n = nrow(r.fam.dat), type = 'z.stat')
```

```
## $type
## [1] "z.stat"
## 
## $test.stat
## [1] 0.2201893
## 
## $P.value
## [1] 0.8257237
```

## References

Adams, D. C. 2014. Quantifying and comparing phylogenetic
evolutionary rates for shape and other high-dimensional phenotypic data.
*Syst. Biol.* 63:166–-177. doi:10.1093/sysbio/syt105

Jetz, W. and R. A. Pyron. 2018. The interplay of past diversification
and evolutionary isolation with present imperilment across the amphibian
tree of life. *Nat. Ecol. Evol.* 2:850–-858. doi:10.1038/s41559-018-0515-5

Juarez, B. H. and D. C. Adams. 2022. Evolutionary allometry of sexual
dimorphism of jumping performance in anurans. *Evol. Ecol.*
36:717–733. doi:10.1007/s10682-021-10132-x

Magallón, S. and M. J. Sanderson. 2001. Absolute diversification
rates in angiosperm clades. *Evolution* 55:1762–-1780. doi:10.1111/j.0014-3820.2001.tb00826.x

Moen, D. S., D. J. Irschick, and J. J. Wiens. 2013. Evolutionary
conservatism and convergence both lead to striking similarity in
ecology, morphology and performance across continents in frogs.
*Proc. R. Soc. B* 280:20132156. doi:10.1098/rspb.2013.2156

Moen, D. S., R. N. Ravelojaona, C. R. Hutter, and J. J. Wiens. 2021.
Testing for adaptive radiation: A new approach applied to Madagascar
frogs. *Evolution* 75:3008–-3025. doi:10.1111/evo.14328
