# Supplementary material for: The radiation continuum and the evolution of frog diversity
Source: Nat Commun. 2023 Nov 4;14:7100. doi: 10.1038/s41467-023-42745-x (PMC10625520; doi:10.1038/s41467-023-42745-x)
Supplement: Supplementary file 10 — Reporting Summary [file 41467_2023_42745_MOESM10_ESM.pdf]

nature portfolio

Corresponding author(s): Daniel S. Moen

Last updated by author(s): Oct 9, 2023

Reporting Summary

Nature Portfolio wishes to improve the reproducibility of the work that we publish. This form provides structure for consistency and transparency in reporting. For further information on Nature Portfolio policies, see our Editorial Policies and the Editorial Policy Checklist.

Statistics

For all statistical analyses, confirm that the following items are present in the figure legend, table legend, main text, or Methods section.

n/a	Confirmed	
☐	☒	The exact sample size (*n*) for each experimental group/condition, given as a discrete number and unit of measurement
☒	☐	A statement on whether measurements were taken from distinct samples or whether the same sample was measured repeatedly
☐	☒	The statistical test(s) used AND whether they are one- or two-sided<br>*Only common tests should be described solely by name; describe more complex techniques in the Methods section.*
☐	☒	A description of all covariates tested
☐	☒	A description of any assumptions or corrections, such as tests of normality and adjustment for multiple comparisons
☐	☒	A full description of the statistical parameters including central tendency (e.g. means) or other basic estimates (e.g. regression coefficient) AND variation (e.g. standard deviation) or associated estimates of uncertainty (e.g. confidence intervals)
☐	☒	For null hypothesis testing, the test statistic (e.g. *F*, *t*, *r*) with confidence intervals, effect sizes, degrees of freedom and *P* value noted<br>*Give P values as exact values whenever suitable.*
☒	☐	For Bayesian analysis, information on the choice of priors and Markov chain Monte Carlo settings
☒	☐	For hierarchical and complex designs, identification of the appropriate level for tests and full reporting of outcomes
☒	☐	Estimates of effect sizes (e.g. Cohen's *d*, Pearson's *r*), indicating how they were calculated

Our web collection on statistics for biologists contains articles on many of the points above.

Software and code

Policy information about availability of computer code

Data collection	We used ImageJ v. 1.52a (Schneider et al. 2012 Nat. Methods) to measure areas from images of frog hands and feet.
Data analysis	All data analyses were conducted in R ver. 4.1.0. Packages tidyr (v. 1.2.0), dplyr (v. 1.0.6), geiger (v. 2.0.7), geomorph (v. 4.0.1), phytools (v. 1.0.1), ape (v. 5.5), vegan (v. 2.5.7), ggplot2 (v. 3.3.5), geometry (v. 0.4.5), hypervolume (v. 3.0.2), patchwork (v. 1.1.1), viridis (v. 0.6.2), scales (v. 1.1.1), hexbin (v. 1.28.2), ggnewscale (v. 0.4.5), tibble (v. 3.1.2), ggrepel (v. 0.9.1), cowplot (v. 1.1.1), phylolm (v. 2.6.2), and ggtree (v. 3.0.4) were used. All data analysis code, including custom scripts written for data analysis and visualization, are permanently archived and available on Zenodo (https://doi.org/10.5281/zenodo.8422404).

For manuscripts utilizing custom algorithms or software that are central to the research but not yet described in published literature, software must be made available to editors and reviewers. We strongly encourage code deposition in a community repository (e.g. GitHub). See the Nature Portfolio guidelines for submitting code & software for further information.

Data

Policy information about availability of data

All manuscripts must include a data availability statement. This statement should provide the following information, where applicable:

- Accession codes, unique identifiers, or web links for publicly available datasets
- A description of any restrictions on data availability
- For clinical datasets or third party data, please ensure that the statement adheres to our policy

All data, including raw intraspecific morphological data, species means, microhabitat states, and phylogenies are available as Supplementary Data. They have also been permanently archived and are available on the Dryad Digital Repository (https://doi.org/10.5061/dryad.hx3ffbggp)127. Data on microhabitats (Supplementary Data 1) were in part gathered from the publicly available web databases AmphibiaChina (http://www.amphibiachina.org/), AmphibiaWeb (http://amphibiaweb.org), Anfibios del Ecuador (https://bioweb.bio/faunaweb/amphibiaweb/), and IUCN (http://www.iucnredlist.org).

Research involving human participants, their data, or biological material

Policy information about studies with human participants or human data. See also policy information about sex, gender (identity/presentation), and sexual orientation and race, ethnicity and racism.

Reporting on sex and gender	This research did not involve human participants, their data, or their biological material.
Reporting on race, ethnicity, or other socially relevant groupings	This research did not involve human participants, their data, or their biological material.
Population characteristics	This research did not involve human participants, their data, or their biological material.
Recruitment	This research did not involve human participants, their data, or their biological material.
Ethics oversight	This research did not involve human participants, their data, or their biological material.

Note that full information on the approval of the study protocol must also be provided in the manuscript.

Field-specific reporting

Please select the one below that is the best fit for your research. If you are not sure, read the appropriate sections before making your selection.

☐ Life sciences ☐ Behavioural & social sciences ☒ Ecological, evolutionary & environmental sciences

For a reference copy of the document with all sections, see nature.com/documents/nr-reporting-summary-flat.pdf

Ecological, evolutionary & environmental sciences study design

All studies must disclose on these points even when the disclosure is negative.

Study description	We collected morphological data on ten functional variables from museum specimens of anurans (frogs and toads). We analyzed these data with phylogenetic comparative methods to calculate rates of multivariate phenotypic evolution and multidimensional morphological diversity for anuran clades. We then estimated net rates of diversification for these clades. We calculated correlations among rates and diversity, then used the rates to define a radiation space. Finally, we assigned clades to this space based on their rates and calculated what proportion of species and morphological diversity each quadrant of this space represented.
Research sample	We sampled 1,234 species of anurans from around the world. Our sample was intended to represent all of anuran history, and specific species were sampled based on accessibility at museum collections and occurrence on the phylogeny of Pyron (2014 Syst. Biol.). We sampled only adult specimens and targeted males (89% of all sampled specimens; 82% of our sampled species were represented only by males). Some of our data came from previously published datasets (Moen et al. 2013 Proc. R. Soc. B; Moen et al. 2016 Syst. Biol.; Moen and Wiens 2017 Am. Nat.).
Sampling strategy	We sampled species based on on accessibility at museum collections and occurrence on the phylogeny of Pyron (2014 Syst. Biol.). Sample size per species was determined by the minimum size to achieve low standard errors in previous studies (e.g., Moen et al. 2016 Syst. Biol.). This was necessary due to the large scale of sampling (32 individual measurements from each of 4,628 specimens across 1,234 specimens from 18 museum collections across the United States). We ideally collected data from 5 individuals per species but often needed to reduce sampling to accommodate museum specimen availability (e.g., only 2 specimens may have been available in all museum collections of the United States).
Data collection	Data were collected by Daniel S. Moen and Gen Morinaga, with assistance from K. Adams, H. Brew, M. Caron, H. Dupire, A. Fery, A. R. Hanna, L. Lacy, E. Mendoza, B. Rae, E. Shore, S. Starr, M. Stevens, A. Vargas, C. Slattery, J. Spicer, A. Van Pelt, S. P. Vijayakumar, M. Wisdom, and A. Zakrzewicz. We used precision calipers to record linear measurements. For area measurements, we photographed

	hands and feet with a Canon EOS 6D camera and 100mm macro lens. We then measured structures in images with ImageJ (Schneider et al. 2012 Nature Methods).
Timing and spatial scale	Data were collected from August 2015 to March 2020, with nearly constant measurement and sampling throughout this time period. Sampling frequency occasionally depended on receipt of shipments from museum collections or visits to those collections. These specimens represent species found around the world, on all continents except Antarctica.
Data exclusions	Morphological data from species that were not on the gene-based phylogeny were excluded from phylogenetic comparative analysis. Moreover, while we collected data from 51 families of anurans, we excluded 8 families for which we only sampled one species. The rationale is that our metric of rate of morphological evolution could not be calculated with just a single species.
Reproducibility	All individuals that measured specimens were trained to reproduce measurements on a core set of specimens. Training ceased once the standard deviation of repeated measurements was within 2% of the pre-established value. Training sessions were replicated three times per week until this benchmark was achieved, after which all attempts at replication were successful. No training data were used as data for the final project (i.e., the data we published with this paper).
Randomization	No experiments were conducted, therefore no randomization was conducted. Species were semi-randomly chosen for sampling, based on availability in museum collections, presence in the phylogeny of Pyron (2014 Syst. Biol.), and a desire to sample equal proportions of species across families.
Blinding	No experiments were conducted, thus blinding was neither necessary nor possible.

Did the study involve field work? ☐ Yes ☒ No

Reporting for specific materials, systems and methods

We require information from authors about some types of materials, experimental systems and methods used in many studies. Here, indicate whether each material, system or method listed is relevant to your study. If you are not sure if a list item applies to your research, read the appropriate section before selecting a response.

Materials & experimental systems

n/a	Involved in the study
☒	☐ Antibodies
☒	☐ Eukaryotic cell lines
☒	☐ Palaeontology and archaeology
☐	☒ Animals and other organisms
☒	☐ Clinical data
☒	☐ Dual use research of concern
☒	☐ Plants

Methods

n/a	Involved in the study
☒	☐ ChIP-seq
☒	☐ Flow cytometry
☒	☐ MRI-based neuroimaging

Animals and other research organisms

Policy information about studies involving animals; ARRIVE guidelines recommended for reporting animal research, and Sex and Gender in Research

Laboratory animals	No laboratory animals were used in this study.
Wild animals	This study did not involve live wild animals.
Reporting on sex	Findings apply primarily to male anurans, as we sampled only adult specimens and targeted males (89% of all sampled specimens; 82% of our sampled species were represented only by males). No analyses separated males from females, primarily because those species for which we sampled females are those in which males and females have highly similar morphology in the variables we measured (i.e., when the males and females were very different, we used that difference to identify males and only sample them). Moreover, previous studies that have statistically tested for differences between males and females under our sampling scheme did not find any significant differences (Moen et al. 2013 Proc. R. Soc. B; Juarez et al. 2020 Evol. Biol.).
Field-collected samples	This study did not involve field-collected samples of live animals.
Ethics oversight	No ethical approval was need for the study. All animals measured for the study came from museum collections.

Note that full information on the approval of the study protocol must also be provided in the manuscript.

Plants

Seed stocks	*Report on the source of all seed stocks or other plant material used. If applicable, state the seed stock centre and catalogue number. If plant specimens were collected from the field, describe the collection location, date and sampling procedures.*
Novel plant genotypes	*Describe the methods by which all novel plant genotypes were produced. This includes those generated by transgenic approaches, gene editing, chemical/radiation-based mutagenesis and hybridization. For transgenic lines, describe the transformation method, the number of independent lines analyzed and the generation upon which experiments were performed. For gene-edited lines, describe the editor used, the endogenous sequence targeted for editing, the targeting guide RNA sequence (if applicable) and how the editor was applied.*
Authentication	*Describe any authentication procedures for each seed stock used or novel genotype generated. Describe any experiments used to assess the effect of a mutation and, where applicable, how potential secondary effects (e.g. second site T-DNA insertions, mosiacism, off-target gene editing) were examined.*
